# Storage and Distribution of Organic Carbon and Nutrients in Acidic Soils Developed on Sulfidic Sediments: The Roles of Reactive Iron and Macropores

**DOI:** 10.1021/acs.est.3c11007

**Published:** 2024-05-14

**Authors:** Changxun Yu, Nguyen Tan Luong, Mohammed E. Hefni, Zhaoliang Song, Eva Högfors-Rönnholm, Sten Engblom, Shurong Xie, Roman Chernikov, Markus Broström, Jean-François Boily, Mats E. Åström

**Affiliations:** † Department of Biology and Environmental Science, Linnaeus University, 39231 Kalmar, Sweden; ‡ Department of Chemistry, Umeå University, 90187 Umeå, Sweden; § Department of Chemistry and Biomedical Sciences, Linnaeus University, 39231 Kalmar, Sweden; ∥ Institute of Surface-Earth System Science, School of Earth System Science, Tianjin University, Tianjin 300072, China; ⊥ Research and Development, Novia University of Applied Sciences, 65200 Vaasa, Finland; # School of Earth Sciences, East China University of Technology, Nanchang 330013, China; ∇ Canadian Light Source, 44 Innovation Boulevard, Saskatoon, Saskatchewan S7N 2 V3, Canada; ○ Thermochemical Energy Conversion Laboratory, Department of Applied Physics and Electronics, Umeå University, 90187 Umeå, Sweden

**Keywords:** acid sulfate soil, macropores, reactive iron, sulfide oxidation, organic carbon storage, nutrients

## Abstract

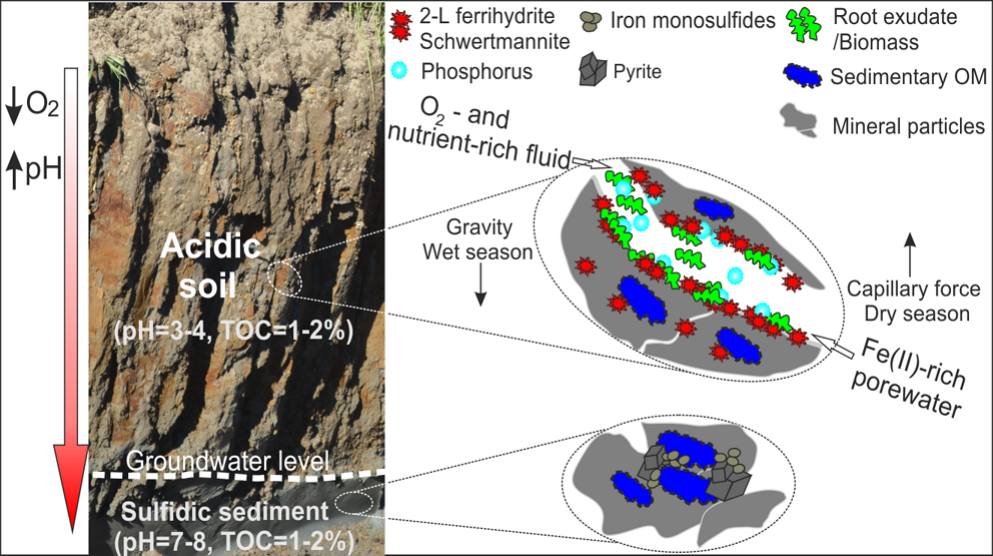

In a boreal acidic sulfate-rich subsoil (pH 3–4) developing on sulfidic and organic-rich sediments over the past 70 years, extensive brownish-to-yellowish layers have formed on macropores. Our data reveal that these layers (“macropore surfaces”) are strongly enriched in 1 M HCl-extractable reactive iron (2–7% dry weight), largely bound to schwertmannite and 2-line ferrihydrite. These reactive iron phases trap large pools of labile organic matter (OM) and HCl-extractable phosphorus, possibly derived from the cultivated layer. Within soil aggregates, the OM is of a different nature from that on the macropore surfaces but similar to that in the underlying sulfidic sediments (C-horizon). This provides evidence that the sedimentary OM in the bulk subsoil has been largely preserved without significant decomposition and/or fractionation, likely due to physiochemical stabilization by the reactive iron phases that also existed abundantly within the aggregates. These findings not only highlight the important yet underappreciated roles of iron oxyhydroxysulfates in OM/nutrient storage and distribution in acidic sulfate-rich and other similar environments but also suggest that boreal acidic sulfate-rich subsoils and other similar soil systems (existing widely on coastal plains worldwide and being increasingly formed in thawing permafrost) may act as global sinks for OM and nutrients in the short run.

## Abstract 2

Our results show that the retention, redistribution, and transformation of reactive Fe phases play a central role in regulating the storage and distribution of organic matter and nutrients (nitrogen and phosphorus) during the formation of acidic soils.

## Introduction

Globally, soil hosts the largest terrestrial reservoirs of organic carbon (OC) and nutrients, such as nitrogen (N) and phosphorus (P).^1,2^ The dynamics and functioning of these reservoirs play pivotal roles in sustaining soil fertility and regulating the levels of greenhouse gases in the atmosphere.^1,3^ Increasing evidence indicates that minerals interact intimately with OC and nutrients, which together with other factors exert a large control over OC storage and nutrient bioavailability/recycling in various soil ecosystems.^4−9^ Reactive iron (Fe) minerals have been shown to play a crucial role in these processes.^10−12^ As such, it is essential to unravel the primary factors and mechanisms that govern the distribution and transformation of reactive Fe minerals as well as their associations/interactions with OC and nutrients in different soil ecosystems.

Subsoil, despite being poor in OC compared to topsoil, has a greater thickness and higher degree of compactness/deformation and thus holds a larger pool of reactive Fe minerals.^13^ These physical and compositional properties enable the subsoil to act as a crucial, yet frequently overlooked, reservoir of terrestrial OC and nutrients.^14,15^ Subsoil also contains a considerable volume of interconnected cracks, fissures, and tubular pores (hereafter collectively referred to as “macropores” or “macropore system”) that vary greatly in three-dimensional geometry, mainly depending on soil structure, fauna activities, and preferential water flow patterns.^16,17^ These macropores serve as hotspots for (bio)geochemical reactions/processes, microbial activities, and the exchange and advection/movement of liquid and gaseous phases,^18−20^ which in turn regulates OC/nutrient distribution, transformation, and storage in subsoil systems.^14,17,20^ During the last two decades, our understanding of the factors and mechanisms controlling OC/nutrient sequestration, transformation, and stabilization in subsoil has greatly expanded.^21−25^ However, it is still unclear how macropores and associated physical/(bio)geochemical reactions/processes could reshape and contribute to the distribution and storage of OC and nutrients in acidic sulfate-rich subsoil systems that are widespread on many coastal plains^26−28^ and certainly increasing in extent in thawing (sub)arctic regions.^29−33^

In this context, acid sulfate (AS) soils, which develop on sulfidic sediments on many low-lying coastal and some inland plains,^27,28,34−37^ are of particular interest. The sulfidic sediments are typically rich in OC and nutrients, and consequently, the subsoil layers of AS soils host large pools of these compounds,^38,39^ in contrast to other mineral soils, whose OC/nutrient stocks are largely stored within the top layers. One characteristic of AS soils is that the (sub)oxic subsoil layers retain abundant reactive Fe as secondary Fe oxyhydroxides (e.g., ferrihydrite) and oxyhydroxysulfates (e.g., schwertmannite and jarosite), in particular on exposed surfaces of hydrologically active macropores.^40−42^ Furthermore, the groundwater table in many of these soils is shallow and thus can vary substantially due to variations in local/regional climato-hydrological conditions. Consequently, the reactive Fe minerals are subject to alternating redox cycles and linked transformation/redistribution processes. It is, however, unclear how these redox cycles and linked processes reshape the large OC and nutrient stocks in the soils. Also, despite that Fe (hydr-)oxides have been widely recognized as key mineralogical controls on OC in acidic soils,^43−46^ the potential and effectiveness of Fe oxyhydroxysulfates in trapping/stabilizing OC under acidic sulfate-rich conditions remained largely unconstrained.

In this study, we combined spectroscopic, chemical, and physical analytical techniques to characterize the mineralogical and chemical properties and reactivities of Fe, OC, and nutrients in an AS soil experimental field. The field is located in the boreal zone, where AS soils are widespread, in particular around the Gulf of Bothnia.^27,36^ The aims were to (i) obtain a detailed and molecular-level understanding of Fe solid-phase speciation and transformation across different AS soil zones and (ii) examine the importance and roles of reactive Fe in mediating the retention/recycling of nutrients (P and N) and the transformation and long-term storage of OC in these soils.

## Materials and Methods

### Study Site and Samples

The study site was the Risöfladan experimental field, located in western Finland (63°2.76′N, 21°42.5′E). The field has been described in detail previously.^47^ It is part of a polder created by subsurface drainage in the 1950s for agricultural cultivation. Under the plow layer (having a pH of 4.1–7.7, due to surface liming), there is a thick oxidized zone that is severely acidic (pH= 3.5–4.0) and heavily cracked, and consists of clayish blocks, whose outer walls are coated by massive yellow-to-brownish precipitates (Figure S1). The oxidized zone turns into a relatively thin transition zone, where pH rises rapidly, underneath which there is a near-neutral anoxic sulfide-rich brackish-water sediment (the soil’s parent material, hereafter referred to as the “reduced zone”). The color of the sediment is black, which is a diagnostic feature of sediments in this region formed during the Littorina Sea and post-Littorina Sea stages of the development of the Baltic Sea.^26^

In August 2018, 1.5–1.7 m deep trenches were excavated at 11 sites across the field (Figure S1 and Table S1). The oxidized zone at each trench was subdivided into three subzones (Figure S1), where the precipitates on exposed macropores (hereafter “macropore surface”) were gathered using a spatula (layer-by-layer until no yellow-to-brownish material was visible), and the underlying (∼0.5 cm thick) clayish layers (hereafter “macropore interior”) were then collected in a similar way. In addition, samples were collected from the reduced zone in one trench, the transition zone in two trenches, and the yellow-to-brownish precipitates at two locations in a ditch draining the field. To prevent oxidation of air-sensitive species in the reduced- and transition-zone samples, 15 mL Falcon tubes were filled with fresh material, after which the capped tubes were wrapped with parafilm and transported in an ice box. In the laboratory, portions of the samples were used for HCl extraction, while the remaining materials were dried in a N_2_ atmosphere for later analyses.

### HCl-Extractable Fe, S, and P

Fresh subsamples (equivalent to ∼1 g of dry material) were extracted with 40 mL of 1 M HCl for 4 h on an orbital shaker at 120 rpm. Thereafter, the suspensions were centrifuged (8000 rpm, 10 min), and the supernatants were filtered through 0.45 μm poly(ether sulfone) membrane filters. The extractants were analyzed immediately for concentrations of Fe(II) via the 1,10-phenanthroline method using spectrophotometry and further for concentrations of Fe, total sulfur (S), and P using inductively coupled plasma optical emission spectroscopy. The analytical precision calculated based on the results of duplicate measurements was better than 2.0%. The 1 M HCl-extractable Fe(III) pools were defined as the differences between the concentrations of Fe and Fe(II). The 1 M HCl extraction was used to recover “reactive” Fe, S, and P pools that are bound to metastable Fe sulfides, carbonates, poorly crystalline Fe (hydr-)oxides and oxyhydroxysulfates,^48−50^ plus a large fraction of jarosite and small fractions of more crystalline Fe oxides (e.g., hematite) and some phyllosilicates (e.g., biotite, illite, and chlorite).^50−52^

### Total Organic Carbon and Total Nitrogen

The transition- and reduced-zone samples, plus 34 samples from the macropore surfaces and corresponding interior counterparts at selected sites, were analyzed for total organic carbon (TOC) and total nitrogen (TN). The TOC and TN analyses were performed on 0.2 g of dried and pulverized subsamples using an ELTRA CS-800 carbon analyzer and an Elementar Rapid N Exceed nitrogen analyzer, respectively. Before the TOC analysis, the subsamples were decarbonated by pretreating with 3 M HCl.

### Hot-Water-Extractable and Acid-Hydrolyzable Organic Carbon

The dried samples analyzed for TOC were also analyzed for concentrations of hot-water-extractable and acid-hydrolyzable OC. Hot-water-extractable and acid-hydrolyzable organic fractions were extracted with MQ water (45 °C and 8 h) and 1 M HCl (room temperature, 4 h), respectively. Hot-water-extractable OC fractions have commonly been considered to be the most labile and readily decomposable OC pool in soils,^53−55^ while acid hydrolysis with 1 M HCl can lead to an efficient extraction of chemically reactive organic components, leaving nonhydrolyzable residues dominated by lignin and related compounds, along with fats, waxes, resins, and suberins.^55−57^ Details on the extraction and determination of the two OC fractions are given in Text S1.

### Thermogravimetric Analysis and Thermal Decomposition of Organic Matter

Thermogravimetric analysis (TGA) was performed on subsamples (both original and pretreated with 3 M HCl to remove inorganic carbon via CO_2_ degassing) from the macropore surfaces and corresponding interior counterparts at three sites plus one sample from the reduced zone. The HCl-treated subsamples (without the removal of supernatant solutions) were dried at 80 °C before being pulverized into powder. For TGA measurement, ∼25 mg pulverized subsample was heated from 50 to 1000 °C at a rate of 10 °C/min under constant air flow using a Q5000 thermogravimetric analyzer. The weight of the material was recorded continuously during the heating process. To further evaluate the thermal stability of the organic matter (OM) in these samples, the fraction of CO_2_ in the exhausted gas from the HCl-treated subsamples was analyzed by an LI-7000 nondispersible infrared CO_2_ analyzer every second using a constant flow of N_2_(g) as a reference gas. The background CO_2_ levels (∼400 ppm), measured from the carrier gas equilibrating with each sample for 10 min at 50 °C, were subtracted from the CO_2_ thermograms. A humic acid reference was treated with 3 M HCl and analyzed in parallel with the subsamples, representing the thermal stability of humic substances in terrestrial ecosystems.

### High-Performance Liquid Chromatography (HPLC) Analysis

The dried samples with TOC data were analyzed in duplicate for abundances of hydroxy radicals (OH^•^) using an HPLC assay developed previously.^58^ The details are given in Text S2.

### Fe X-ray Absorption Spectroscopy (XAS)

Iron K-edge XAS was used to probe solid-phase Fe speciation in different materials from the field, including dried samples of the reduced zone (*n* = 2), transition zone (*n* = 2), ditch precipitates (*n* = 2), and macropore surfaces and corresponding interior counterparts from seven sites (*n* = 14). The XAS data were recorded on the BioXAS-Spectroscopy beamline of the Canadian Light Source and the Balder beamline of the MAX IV laboratory (for details, see Text S3).

To aid interpretation of the experimental XAS data, a total of 18 Fe reference spectra were compiled, representing a wide range of Fe coordination environments that possibly occur in the samples (for details, see Table S2). The XAS spectra were processed and analyzed, following standard procedures, using the Demeter and SIXpack software packages.^59,60^ The extended X-ray absorption fine structure (EXAFS) spectra were extracted by using a cubic spline function. Principal component analysis in combination with target transformation testing was applied to the *k*^3^-weighted EXAFS spectra, in order to identify the reference spectra most suitable for quantifying Fe speciation in the samples via a linear combination fitting (LCF) approach under the guidance of F-tests. The detailed information is given in Text S4.

## Results

### Iron, Sulfur, and Phosphorus

**Figure 1:**
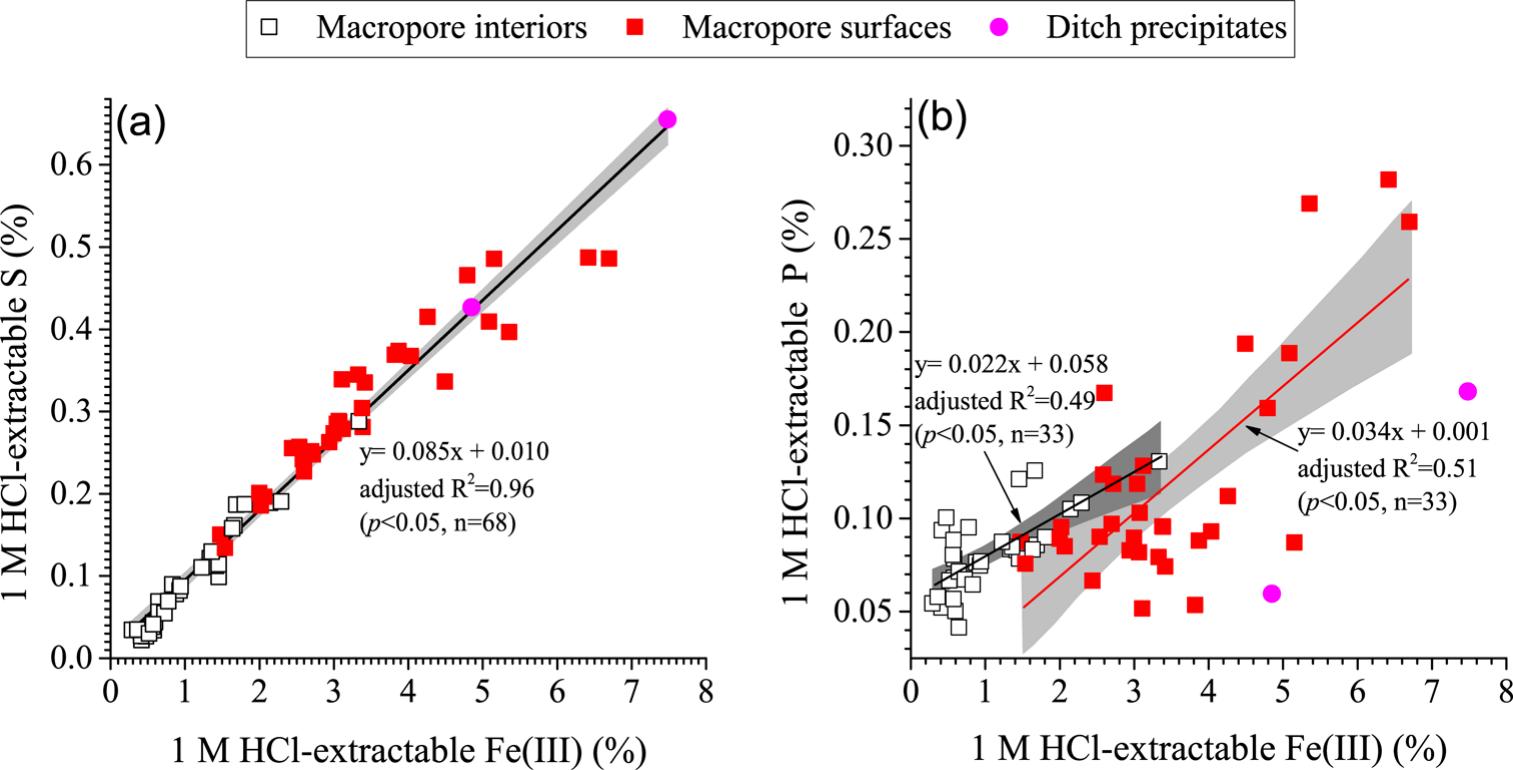
Concentrations of 1 M HCl-extractable Fe(III) versus those of 1 M HCl-extractable S and P on macropore surfaces and their interior counterparts in the oxidized zone. Two samples of precipitates from a ditch were also included for comparison. The black solid line in panel (a) is the best linear regression fit for the concentrations of 1 M HCl-extractable S and Fe(III) in all of the samples, while the red and black line in panel (b) represent the best linear regression fit for the concentrations of 1 M HCl-extractable P and Fe(III) on macropore surfaces and in their interior counterparts, respectively. The shaded areas represent the 95% confidence intervals for the fits. The standardized residuals of the fits overall followed a normal distribution (reflected by an (semi)symmetrical shape of the histograms and an overall agreement of the observed and expected data in the normal probability plots, Figures S7–S9), validating the reliability of the linear regression models.

In the transition- and reduced-zone samples, the 1 M HCl-extractable Fe pools occurred exclusively as ferrous Fe, with concentrations of 0.31–0.32 and 0.94–1.04%, respectively (Table S1). However, the concentration of 1 M HCl-extractable S was approximately 2 orders of magnitude higher in the reduced zone (1.20–1.33%) than in the transition zone (0.01–0.02%) (Table S1). Consistent with the well-aerated conditions in the ditch and the macropores within the oxidized zone, ferric Fe strongly dominated the 1 M HCl-extractable Fe pools in these systems (Table S1). Moreover, the concentrations of 1 M HCl-extractable Fe(III) on the macropore surfaces (1.47–6.70%) were strongly elevated relative to their interior counterparts (0.29–3.34%) (Figure 1a and Table S1), with a mean difference (*p* < 0.05) of 2.38 ± 0.25% (Table 1). This is in line with the fact that the former was covered by distinctive yellow-to-brownish precipitates (Figure S1). The 1 M HCl-extractable Fe(III) and S were also strongly correlated within the macropore system (Figure 1a and Tables 1 and S1).

**Table 1:** Results for the Tamhane’s T2 Post Hoc Test of Significance of Differences in 1 M HCl-Extractable Fe(III) and S, TOC, TN, atomic ratios of TOC/TN, pH of Hot Water Extracts, Hot-Water and 1 M HCl-Extractable DOC, and Hydroxy Radicals between the Macropore Surfaces (MSs), the Macropore Interiors (MIs), and the Transition and Reduced Zones (TRZ)

					95% confidence interval	
variable	sample	mean difference	std. error	*p* value	lower bound	upper bound
1 M HCl-extractable Fe(III) (%)	MSs vs MIs	2.375^a^	0.251	0.000	1.754	2.997
	MSs vs TRZ	3.458^a^	0.222	0.000	2.898	4.017
	MIs vs TRZ	1.082^a^	0.118	0.000	0.784	1.381
1 M HCl-extractable S (%)	MSs vs MIs	0.217^a^	0.020	0.000	0.168	0.266
	MSs vs TRZ	–0.331	0.361	0.811	–2.070	1.407
	MIs vs TRZ	–0.549	0.361	0.536	–2.289	1.192
1 M HCl-extractable P (%)	MSs vs MIs	0.036^a^	0.011	0.006	0.009	0.064
	MSs vs TRZ	0.054^a^	0.011	<0.001	0.027	0.081
	MIs vs TRZ	0.018^a^	0.005	0.009	0.004	0.031
TOC (%)	MSs vs MIs	–0.282^a^	0.049	0.000	–0.406	–0.158
	MSs vs TRZ	–0.352^a^	0.083	0.032	–0.661	–0.042
	MIs vs TRZ	–0.069	0.083	0.830	–0.379	0.240
TN (%)	MSs vs MIs	0.000	0.000		0.000	0.000
	MSs vs TRZ	–0.075	0.025	0.163	–0.196	0.046
	MIs vs TRZ	–0.075	0.025	0.163	–0.196	0.046
atomic TOC/TN ratios	MSs vs MIs	–0.972^a^	0.348	0.043	–1.916	–0.029
	MSs vs TRZ	–0.219	0.410	0.942	–1.565	1.128
	MI vs TRZ	0.753	0.426	0.325	–0.597	2.104
pH of hot water extracts	MSs vs MIs	0.411^a^	0.131	0.011	–0.742	–0.080
	MSs vs TRZ	–2.013^a^	0.180	0.000	–2.621	–1.406
	MIs vs TRZ	–1.602^a^	0.178	0.001	–2.213	–0.992
hot-water-extractable DOC (%)	MSs vs MIs	–0.035	0.015	0.073	–0.074	0.003
	MSs vs TRZ	–0.070	0.022	0.088	–0.154	0.013
	MIs vs TRZ	–0.035	0.023	0.479	–0.114	0.045
1 M HCl-extractable DOC (%)	MSs vs MIs	0.494^a^	0.044	0.000	0.384	0.606
	MSs vs TRZ	0.501^a^	0.050	0.000	0.358	0.644
	MIs vs TRZ	0.006	0.044	0.999	–0.133	0.146
hydroxy radicals (nmol/kg)	MSs vs MIs	–62.49^a^	13.01	0.000	–95.73	–29.24
	MSs vs TRZ	–78.34	20.88	0.070	–165.4	8.72
	MIs vs TRZ	–15.86	22.87	0.890	–95.23	63.52

a The mean difference is significant at the 0.05 level.

The Fe XANES and EXAFS spectra are presented in the Supporting Information (Text S5) and the results of the LCF-EXAFS analysis are given in Table 2. The key results are (i) Fe-bearing silicates (mainly hornblende/biotite and illite) occurred in all samples, with the highest fractional amounts in the macropore interiors and reduced and transition zones; (ii) ferrihydrite and schwertmannite generally dominated the secondary nonsilicate Fe pools in the samples from the oxidized zone, and in the ditch precipitates, there was also abundant jarosite; and (iii) sulfide minerals, but no secondary Fe(III) phase, occurred in the transition and reduced zones.

**Table 2:** Solid-Phase Speciation of Fe in Selected Samples from the AS Soil Field, Quantified by Linear Combination Fitting of *k*^3^-Weighted EXAFS Spectra (*k* = 2-12 Å^–1^)^a^,^b^,^c^

	sample	Hbl^d^	Bt^d^	Chl	Ill	Aq. Fe(II)	Fe(II)-HA	FeS	Py	2-L Fh	Goe	Fe(III)-HA	Jrs	Sch	*R*-factor^e^
macropore surfaces	A_–surf-1_		10	7						45		17	5	17	0.0431
	A_–surf-2_		8							59		21		12	0.0261
	A_–surf-3_			10	28					51				10	0.0051
	F_–surf-3_	13								57		29			0.0366
	I_–surf-1_				49									51	0.0268
	I_–surf-3_				23	12				46				20	0.0210
	J_–surf-1_		13		20					29				39	0.0159
macropore interiors	A_–int-1_		27		43					31					0.0293
	A_–int-2_		15		43					41					0.0113
	A_–int-3_		34		20					22		24			0.0153
	F_–int-3_	38			36					27					0.0189
	I_–int-1_	36			8							21		34	0.0572
	I_–int-3_		16		34					27				23	0.0296
	J_–int-1_	30			37					33					0.0652
ditch	ditch-_precip-1_		17		21					19			17	26	0.0159
	ditch-_precip-2_				20					17			37	26	0.0236
transition zone	F_–trans-1_	41			30	12			17						0.0852
	J_–trans-1_	44			28		13		15						0.0415
reduced zone	A_–red-1_		29		60			9	2						0.0399
	A_–red-2_		40		45			10	5						0.0328

a The component sums were normalized to 100% (initial range: 85–110%).

b Hbl = hornblende; Bt = biotite; Chl = chlorite; Ill = illite; Aq. Fe(II) = aqueous Fe(II); Fe(II)-HA = Fe(II)-cid; Py = pyrite, 2-L Fh = 2-line ferrihydrite; Goe = goethite; Fe(III)-HA = Fe(III)-sorbed humic acid; Jrs = jarosite; Sch = schwertmannite.

c The subscripts “surf” or “int” refer to macropore surface and interior, respectively, while “red”, “trans”, and “precip” refer to reduced zone, transition zone, and ditch precipitate, respectively. The subscript numbers mark the three subzones (as shown in Figure S1) at each sampling trench.

d Due to the similarities in the EXAFS spectra of these two reference minerals (see Figure S4a), the predicted fractional amounts for these minerals are uncertain.

e *R*-factor = ∑((data – fit)^2^/∑data^2^).

**Figure 2:**
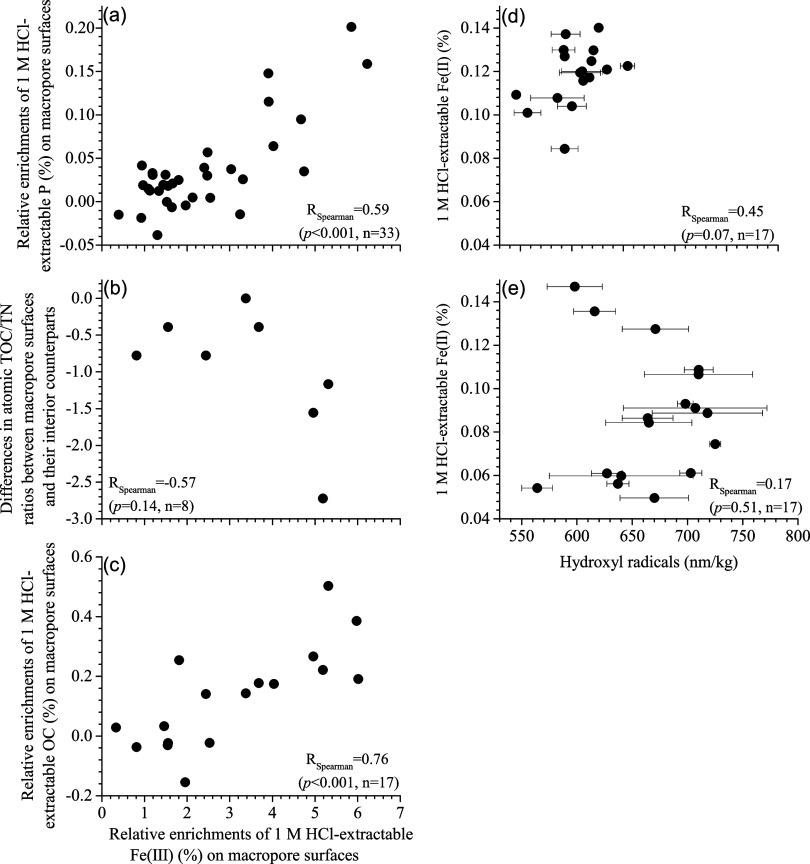
Correlations between relative enrichments of 1 M HCl-extractable Fe(III) on macropore surfaces (with respect to their interior counterparts) and several other parameters (a–c); and the abundances of hydroxy radical versus 1 M HCl-extractable Fe(II) for the macropore surfaces (d) and their interior counterparts (e). Relative enrichments of 1 M HCl-extractable Fe(III), TOC, and P on macropore surfaces are calculated as the differences in the 1 M HCl-extractable concentrations of these variables between macropore surfaces and their interior counterparts.

The concentrations of 1 M HCl-extractable P in the transition- and reduced-zone samples were low and generally stable (0.05–0.07%), and in the macropore interiors overall, only slightly higher, ranging from 0.04 to 0.13% (Figure 1b, Tables 1 and S1). In contrast, on the macropore surfaces, this P fraction was (i) considerably higher but more variable (up to 0.28%) (Figure 1b, Tables 1 and S1); (ii) significantly correlated to 1 M HCl-extractable Fe(III) (Figure 1b); and (iii) enriched relative to the interior counterparts by up to 0.2%, in a manner that correlated to the corresponding relative enrichments of 1 M HCl-extractable Fe(III) (Figure 2a). Similarly, in one of the ditch precipitates, the P concentration was high (Figure 1b).

### Total Organic Carbon and Total Nitrogen

The contents of TOC on the macropore surfaces varied from 0.9 to 1.5%, being significantly (*p* < 0.05) lower than the interior counterparts (1.1–1.6%) both at individual sampling sites and across the field. They were also lower than in the samples from the transition and reduced zones (1.3–1.6%) (Tables 1 and S3). In contrast, the macropore surfaces and interior counterparts had the same TN content (0.3%), similar to those of the transition- and reduced-zone samples (0.3–0.4%) (Tables 1 and S3). As a result, the atomic TOC/TN ratios for the macropore surfaces (4.3–5.8) were slightly but significantly lower than those for the interior counterparts (4.3–6.2) (Tables 1 and S3). The TOC/TN ratios (both the absolute values and the differences between the macropore surfaces and interior counterparts) were inversely correlated to the concentrations of 1 M HCl-extractable Fe(III) (Figure S5a) and the relative enrichments of this Fe fraction on the macropore surfaces (Figure 2b).

### Chemical Fractionation and Thermal Stability of Organic Carbon

The concentrations of hot-water-extractable dissolved organic carbon (DOC) in the samples from the macropore surfaces and interiors varied from 0.04 to 0.20% and from 0.03 to 0.18%, respectively (Table S3), and were statistically indistinguishable both from each other and from the transition- and reduced-zone samples (0.08–0.16%). Furthermore, these represented only relatively small fractions of the TOC pools (Tables 1 and S3). In contrast, 1 M HCl released and solubilized substantially higher proportions of the TOC pools (Table S3), in particular from the macropore surfaces that hold, on average, ∼0.5% more 1 M HCl-extractable DOC than other samples (Table 1). The concentrations of this organic fraction in the macropores (both surfaces and interiors) and its enrichment on the macropore surfaces relative to the interior counterparts were significantly correlated with those of the corresponding 1 M HCl-extractable Fe(III) (Figure 2c and S5b).

**Figure 3:**
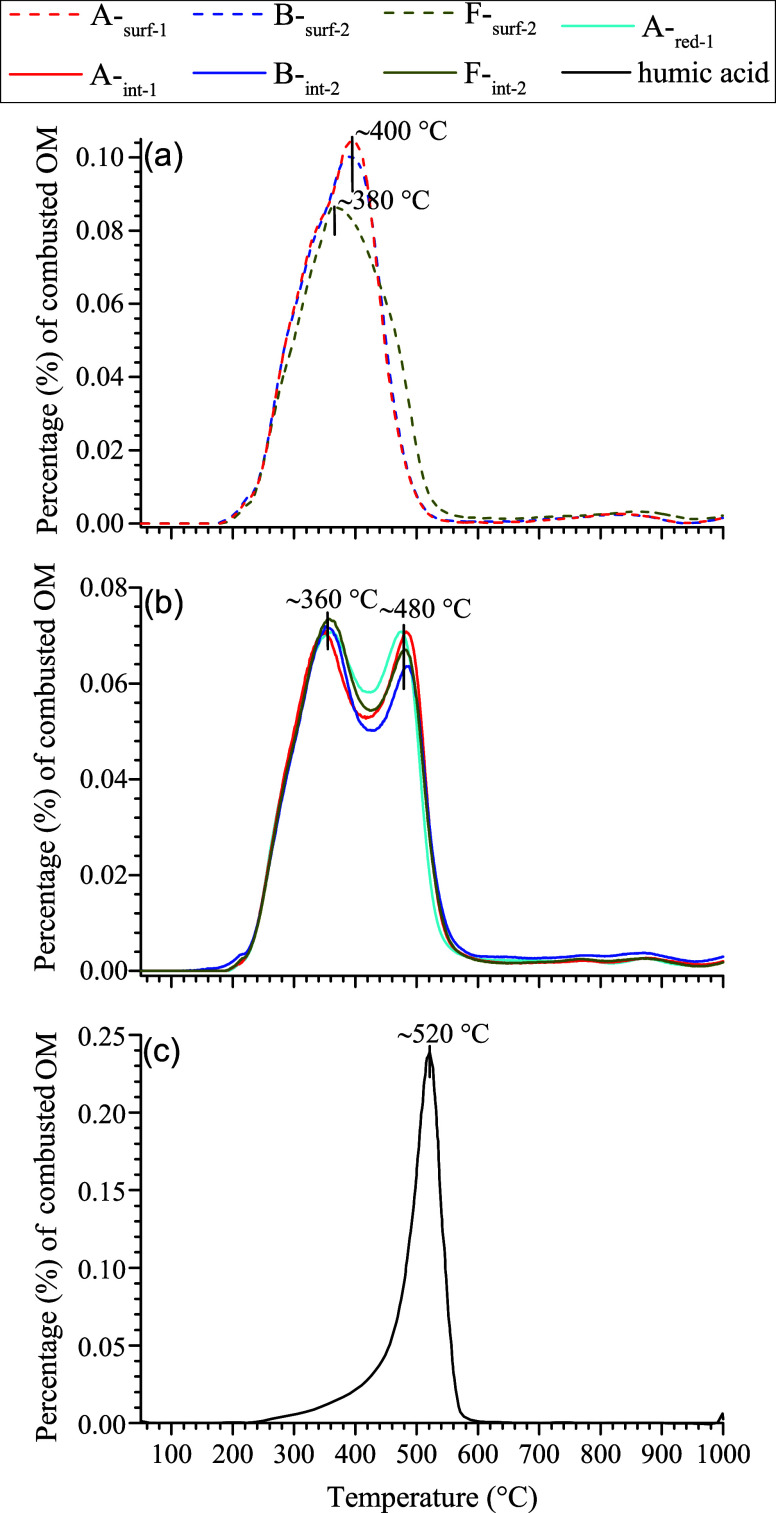
Relative proportions (%) of thermally combusted organic matter (OM) in selected samples as a function of temperature. The fractional amounts of combusted OM (measured as CO_2_ at 0.167 °C/s) were renormalized to 100%. The samples from the macropore surfaces are shown in (a), while those from the interior counterparts plus one sample from the reduced zone in (b). The samples were decarbonated by repeated treatments with 3 M HCl. The data for a humic acid reference (c) that was treated in the same way as the samples was shown for comparison.

During ramped combustion of decarbonated samples, intramolecular OC bonds and/or intermolecular organomineral OC bonds with increasing strengths are expected to successively break, producing volatile OC fragments that are continuously oxidized and quantified as CO_2_ downstream (known as a CO_2_ thermogram). The CO_2_ thermograms obtained for three soil samples of macropore surfaces displayed one broad peak at ∼380 or ∼400 °C (Figure 3a), and the vast majority (∼95%) of the thermally oxidizable OM was recovered at temperatures below 500 °C. In contrast, the CO_2_ thermograms for the interior counterparts and one reduced-zone sample were characterized by two peaks of similar shape and intensity, centering at ∼360 and ∼480 °C, respectively (Figure 3b). Additionally, the ramped combustion at temperatures below 500 °C recovered significantly lower fractions (∼86%) of the total thermally oxidizable organic pools in these samples.

### Hydroxy Radicals

The abundances of OH^•^ on the macropore surfaces ranged from 564 ± 14 to 725 ± 9 nmol/kg, and were, on average, ∼63 ± 13 nmol/kg (*p* < 0.05) and 78 ± 21 nmol/kg (*p* = 0.07) lower than those in the macropore interiors and the samples from the reduced and transition zones, respectively (Tables 1 and S3). The OH^•^ abundances were only weakly correlated to the concentrations of 1 M HCl-extractable Fe(II) on the macropore surfaces (Figure 2d,e), and they displayed no correlation with other variables for the macropore surfaces and interiors, such as TOC, water-extractable DOC, and 1 M HCl-extractable Fe(III) concentrations.

### TGA Weight Losses and Peaks

**Figure 4:**
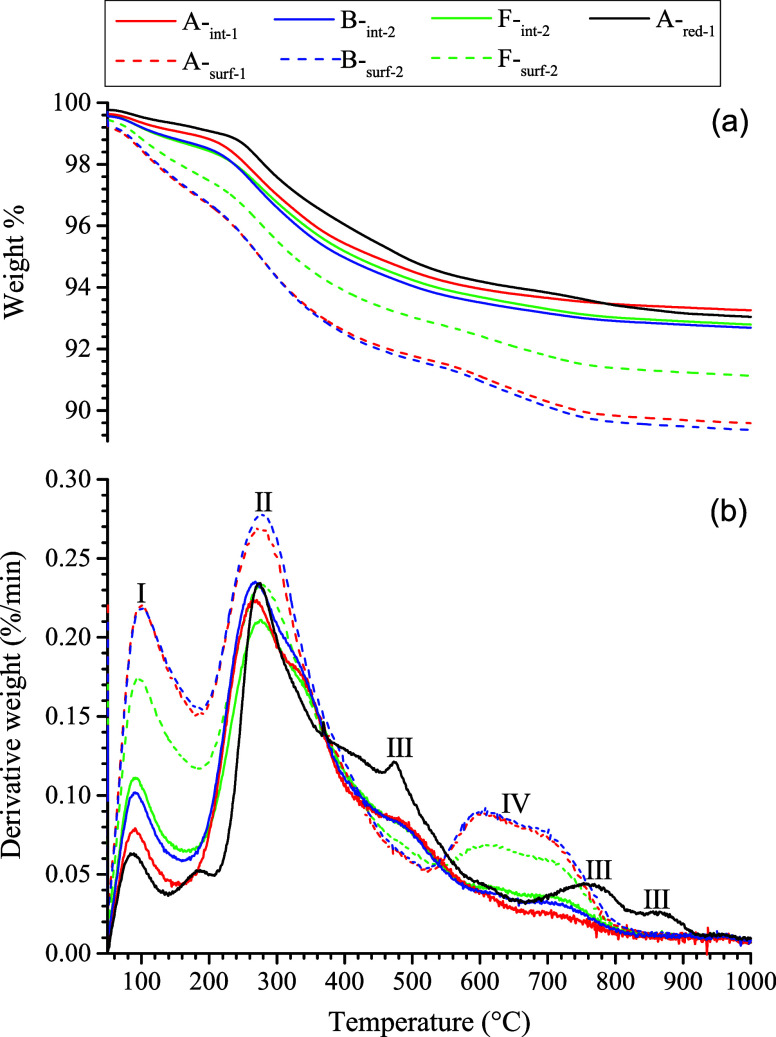
TGA curves showing temperature-dependent change in weight percentage (a) and their derivatives (b) for the samples of macropore surfaces and interior counterparts at three sites plus one sample from the reduced zone. The peaks marked in (b) were interpreted to mainly reflect the weight loss of free and surface-sorbed water (I), dehydration and dehydroxylation of structural H_2_O/OH (II), thermal oxidation of sulfides and subsequent SO_2_ outgassing (III), and loss of structural SO_4_ (IV) primarily bound to Fe oxyhydroxysulfates, e.g., schwertmannite.

In comparison with the interior counterparts and reduced-zone samples, the samples from the macropore surfaces had a much greater weight loss (∼9–10%, Figure 4a), with stronger weight loss peaks between 80–100, 270–280, and 600 and 700 °C, respectively (Figure 4b). These features were interpreted to reflect the occurrence of abundant secondary minerals on the macropore surfaces (e.g., ferrihydrite and schwertmannite as revealed by the LCF-EXAFS analysis and 1 M HCl extraction; Table 2 and Figure 1a). The reduced-zone sample displayed three additional notable peaks, centering around 480, 770, and 880 °C, respectively (Figure 4b), that were likely linked to thermal decomposition of Fe-sulfide minerals. Detailed interpretation of these weight loss peaks is given in Text S6.

## Discussion

### Iron Distribution and Mineralogical Transformations

The LCF-EXAFS results (Table 2) predicted that pyrite and metastable Fe sulfides (modeled as FeS) coexisted in the reduced zone and trapped similar amounts of the reduced S pools, while pyrite was the only detectable iron-sulfide mineral in the transition zone. These features are consistent with previously identified distribution of reduced S phases across typical AS soil profiles in the study region, that is, (i) the S pool in the reduced zone is overall equally distributed between acid volatile sulfur (bound to metastable iron sulfides) and pyrite-S, (ii) the quantities of pyrite-S strongly increase at the expense of acid volatile sulfur close to the boundary between the reduced and transition zones, and (iii) pyrite-S become the predominating reduced S phase in the central and upper parts of the transition zone.^36,61^ The (near-)complete replacement of metastable Fe sulfides by pyrite in the transition zone is a striking feature and might be attributed to two processes cross-linked by elemental S. First, metastable iron sulfides at the base of the transition zone are rapidly oxidized upon contact with percolating soil solutions with O_2_ and other oxidants (e.g., Fe^3+^ and nitrate), producing elemental S as the initial oxidation product under the near-neutral pH conditions.^62,63^ Second, the produced elemental S reacts with solid or aqueous FeS clusters, triggering the formation of pyrite through the polysulfide pathway.^64,65^ Since pyrite oxidation is slow and involves many potential rate-limiting steps, the (near)complete replacement of metastable Fe sulfides by pyrite in the transition zone significantly prolongs the acidification and associated biogeochemical processes and chemical leaching in boreal AS soils.

The macropore surfaces had consistently low pH values (mostly between 3.4 and 4.0; Table S1), consistent with typical active AS soils in the boreal zone.^35,66−68^ The results of previous studies with samples from one site in the same field revealed strong concomitant enrichments of both near-total as well as 1 and 4 M HCl-extractable fractions of Fe and S on macropore surfaces relative to the interiors of solid columnar blocks.^40,41^ This should be a widespread feature for the whole experimental field given that the macropore surfaces sampled from all 11 sites across the field were significantly and concomitantly enriched with 1 M HCl-extractable Fe and S fractions relative to the interior counterparts (Tables 1 and S1). As predicted by the LCF-EXAFS analysis, the 1 M HCl-extractable Fe and S fractions in the samples from the macropore surfaces and interiors were largely bound to ferrihydrite and schwertmannite (Table 2). Even though schwertmannite was predicted to only occur in 2 out of the 7 interior samples (Table 2), it might despite this occur in most of the interior samples as a minor Fe phase, considering (i) the relatively large uncertainties (±3–5%) and detection limit (5%) of the LCF approach in quantifying Fe speciation in natural samples,^69−71^ and (ii) the significant and strong linear correlation between the 1 M HCl-extractable Fe and S fractions, with molar Fe(III)/S ratios (15th-75th percentile: 6.3–8.9) mostly within the range reported for natural schwertmannite,^72−75^ in all the interior samples (Figure 1a). Given the widespread occurrence and massive surface areas of the macropores in the oxidized zones of the AS soils on the experimental field and elsewhere in boreal AS soils, the Fe hydroxides and oxyhydroxysulfates on the macropores should trap large quantities of mobile Fe pools released by pyrite oxidation and pH-promoted weathering of Fe-bearing phyllosilicates during the formation and ripening of AS soils. It is well-documented that, in contrast to a large number of other metals, Fe is lost to only a limited extent, and can even accumulate in the oxidized zones of typical boreal AS soil profiles.^36,66,68,76^ As elaborated previously,^40^ the accumulation of Fe hydroxides and oxyhydroxysulfates on macropore surfaces was likely further contributed by continuous oxidation of dissolved Fe(II) species, diffused locally from the interiors of columnar blocks and supplied via evaporation-driven capillary movement of soil porewater or moisture from underlying transition zones. The relevance of the latter process is further supported by abundant aqueous and organically complexed Fe(II) in the transition zone samples (Table 2). Collectively, these results and features suggest that the oxidation of pyrite in the transition zone is a driving factor for the formation of boreal AS soils, as it not only results in severe soil acidification but also, in conjunction with other factors or processes, creates favorable conditions for the formation and retention of abundant Fe hydroxides and oxyhydroxysulfates, particularly on the macropore surfaces in the oxidized zone.

### Impact of Reactive Iron on Phosphorus Retention

The reduced zones of the AS soils in the study region have been reported to commonly contain 0.05–0.10% *aqua regia*-extractable P.^77^ These concentrations are typical for postglacial fine-grained sediments distributed on the coastal plains of western Finland^78^ and also fall within the range reported for the reduced zones of active AS soils (0.03–0.30%, median = 0.08%) along the Swedish coast.^66^ As compared to the reduced zones, the oxidized zones of the AS soils in those studies had *aqua regia-*extractable P concentrations that ranged from “not significantly depleted” (down to 0.03%) to “strongly enriched” (up to 2.37%).^66,77^ The strong P enrichments cannot be counterbalanced by P loss from other soil zones and thus were assumed to reflect immobilization/retention of external P, that is, excess P derived from phosphate-bearing fertilizers in the plow layer.^77^ A previous study showed that (i) 1 M HCl extracted substantial proportions of the *aqua regia*-extractable P pools (26–88%, median = 64%) in the oxidized zones of Swedish coastal AS soils and (ii) the extractabilities were closely related to those of Fe(III).^66^ The close association between 1 M HCl-extractable P and Fe(III) is also evident for the oxidized zones of the studied AS soil field (Figures 1b and 2a). Since the surfaces of schwertmannite and/or ferrihydrite enriched on the macropore surfaces are positively charged under acidic conditions prevailing in the oxidized zones, they have the potential to trap large amounts of PO_4_^3–^ via the formation of strong inner-sphere surface complexes.^79−81^ Furthermore, schwertmannite can incorporate PO_4_^3–^ into its structure by substituting PO_4_^3–^ for sulfate.^82^ The P trapped by these two processes is expected to have relatively high stability, as supported by the fact that extraction with ammonium acetate (pH = 4) only led to marginal removal of P in the oxidized zones of AS soils from our study region.^77^ The heterogeneous distribution of schwertmannite and ferrihydrite (Table 2) and their intricate interactions with P under varying local physicochemical conditions across different macropores also explain the wider range of 1 M HCl-extractable P concentrations on the macropore surfaces compared to their interior counterparts (Figure 1b). Taken together, these results suggested that excess anthropogenic P added to boreal AS soil farmlands is effectively trapped and stabilized by its intimate interactions with reactive Fe(III) phases (iron oxyhydroxides and oxyhydroxysulfates) in the oxidized zones, in particular, on macropores, whose surfaces are coated with abundant reactive Fe(III) phases and directly interact with percolating P-bearing fluids.

### Factors Influencing the Stocks and Distribution of Organic Matter and Nitrogen

The reduced zone of the AS soil field contained high levels of TOC (1.4–1.6%) and TN (0.3–0.4%) (Table S3). These results support previous findings that the reduced zones of boreal AS soils hold strongly elevated concentrations and stocks of organic C and total/mineral N, in comparison with the parent materials (equivalent to the reduced zones of AS soils) for nearby non-AS soils.^38,39,67,83^ For example, it was reported that the reduced zone of AS soils in the Viikki research farmland on the coast of the Gulf of Finland contained 2.67 ± 0.01% TOC, 0.29 ± 0.004% TN, and 13.3 ± 1.97 mg/kg NH_4_^+^–N (the dominating mineral nitrogen species) and stored ∼110 Mg organic C, ∼15 Mg total N, and ∼330 kg mineral N per hectare.^39,83^ The concentrations and estimated stocks were approximately 3–10 times higher than those for the parental materials of other soils in the same farmland.^39,83^ Thus, it is evident that the reduced zones in typical boreal AS soils are characterized by high levels and stocks of organic C and total/mineral N, which are likely attributed to the high biological production during the deposition of these sediments under brackish-water conditions (Baltic Sea) in the Holocene, and subsequent development/persistence of strictly anaerobic conditions favoring the preservation of OM, accompanied by slow mineralization and concomitant buildup of NH_4_^+^–N.^39^

The transition zone and macropore interiors (representing a majority of the oxidized zone) of the AS soil field also contained high levels of TOC and TN (Table S3), in line with the results of previous studies, showing that boreal AS soils hold substantial TOC and TN stocks in the oxidized and transition zones,^38,39^ in sharp contrast to ordinary mineral soils, where TOC and TN stocks are largely stored in the thin cultivated/rooted (sub)surface layers.^84−86^ Our data also show that the TOC pools in the macropore interiors consist of large proportions of an acid-hydrolyzable organic fraction (22–66% with a median of 45%, *n* = 17), similar to those reported for acidic agricultural, forest, wetland, and grassland soils (7–85% with a median of 39%, *n* = 24) with a silty/sandy loam texture.^87−90^ Since the acid-hydrolyzable organic fractions in the macropore interiors were extracted with less-concentrated HCl at lower temperatures as compared to the previous studies (6 M HCl and 80–120 °C), they were very likely composed of organic compounds of higher chemical reactivities as compared to those defined by the previous studies.^87−90^ The formation/occurrence of abundant reactive Fe(III) phases in the macropore interiors should have contributed to the retention of these organic compounds (via surface sorption and physical entrapments),^10,91,92^ as evidenced by the positive correlation between the concentrations of the acid-hydrolyzable organic fractions and coextracted reactive Fe(III) (Tables S1, S3 and Figure S5b). It is also evident that the macropore interiors of the AS soil field contained higher proportions of hot-water-extractable organic fractions (2.9–14% with a median of 4.4%, *n* = 17) than those reported for acidic silty loam soils (0.25–1.4% with a median of 0.76%, *n* = 35) that had similar pH values (4.2–5.9) and were extracted with MQ water at comparable temperatures (40 or 50 °C).^93−95^ These results suggest that the OM in the macropore interiors (and other similar parts of the oxidized zone) possess high chemical reactivities, which is further supported by its significantly higher reactivity toward thermal decomposition as compared to the humic acid reference (Figure 3b,c). Our results also show that the OM fractions in the reduced/transition zone and macropore interiors not only had similar total contents (Table 1) but also exhibited similar chemical composition and characteristics, as reflected by statistically indistinguishable TOC/TN ratios, hot-water extractability, and 1 M HCl extractability (Tables 1 and S3), as well as almost identical CO_2_ thermograms (Figure 3b). As the TN pools in boreal AS soils are near-quantitively bound to OM,^38,83,96^ the TOC/TN ratios can be assumed to represent the carbon to nitrogen ratios in the total OM fractions. Taken together, these features suggest that most of the OM (and its associated carbon and nitrogen) in the oxidized/transition zone were largely inherently derived from the sulfidic sediment (and thus of sedimentary origins) and that the formation/occurrence of Fe oxyhydroxides and oxyhydroxysulfates in the oxidized zone retained most of the sedimentary OM pools without a significant loss or chemical fractionation.

The CO_2_ thermograms for the three samples from macropore surfaces displayed single broad peaks at low temperatures (380–400 °C, Figure 3a), differing significantly from their interior counterparts (Figure 3b). These features suggest that the sampled macropore surfaces were loaded with distinctly different organic compounds with overall lower resistances to thermal decomposition and, presumably, higher chemical reactivities.^97^ This is further supported by the fact that relative to their interior counterparts, the macropore surfaces contained significantly higher proportions of acid-hydrolyzable and thus chemically reactive organic fractions (Tables 1 and S3). These features collectively suggest that a large part of the organic loads on the macropore surfaces was composed of more labile organic compounds than their interior counterparts. These labile organic compounds could be derived from several sources, including the plow layer, biological activity within the macropores (e.g., root exudates and microbial biomass/bioproducts), and a mobilizable fraction of the inherent OM in the macropore interiors. The strong positive correlations between the accumulations of reactive Fe(III) and acid-hydrolyzable organic fractions on macropore surfaces (Figures 2c and S5b) suggest that these reactive Fe(III) phases (largely as ferrihydrite and schwertmannite, Table 2) have acted as efficient traps of organic compounds that were formed, dissolved, and transported within the macropores of the soils. Furthermore, given iron oxyhydroxides tend to preferentially retain aromatic- and carboxylic-rich organic compounds of high molecular weight via coprecipitation and surface adsorption,^92,98−100^ it is likely that a portion of the labile organic compounds was not trapped, but remained in solution and was eventually leached via the drainpipes.

### Environmental Implications

Our data provide strong evidence that, during the development of the studied AS soil over the 70 years they have existed, sulfide oxidation and associated processes have not induced any noticeable changes in the stock, composition, and reactivity of the OM pool inherited from the sulfidic sediment. This points to the high stabilization potential of the inherent sedimentary OM pool in such soils. However, frequent macropores with abundant secondary Fe(III) minerals were found to trap large amounts of OM and nutrients derived most likely primarily from the vegetated layers. The associations of these secondary Fe(III) minerals with OM can retard their transformation to more crystalline and thermodynamically stable forms,^101−103^ which in turn favor the storage and retention of soil OM and nutrients. It has been estimated that AS soils cover 17–24 million hectares globally,^104^ but the area may be considerably larger. For example, for the coastal plains bordering the Baltic Sea, where the study site is located, old estimates are much lower than the most recent ones.^26,27,34,105−107^ Thus, the results of this study are globally relevant, highlighting the great potential of AS soils as global sinks for both carbon and nutrients, particularly over short-term periods.

To minimize acid and metal leaching from AS soils, it is becoming a common practice to raise the groundwater level or entirely inundate the oxidized zone of the soils.^108−110^ As the macropores in the oxidized zone are largely covered with reactive Fe(III) phases (dominated by 2-line ferrihydrite and schwertmannite as found here) in association with labile OM and nutrients, these macropores can be quickly turned into microbially driven reductive systems during episodic or permanent waterlogging. Although this is beneficial for minimizing acid and metal leaching from AS soils, it could lead to strong increases in the exposure of labile OM to decomposers (and associated production of greenhouse gases) and loads of nutrients and Fe^2+^ in soil water and thus, ultimately, drainage waters.

There is growing evidence that intensified thaw/degradation of permafrost across many terrains in the (sub)arctic regions is frequently accompanied by the liberation of massive acidic sulfate-rich drainages due to the oxidation of sulfidic shales and glacial tills that were previously frozen underground or covered by glaciers.^29,31−33,111,112^ The results of our study suggest that, during the oxidative weathering of these (sub)arctic sulfidic materials, most of the OM may remain intact at least in the short term. The production and outflow of the acidic drainages also result in massive formation of Fe(III) hydroxides and oxyhydroxysulfates (identified/modeled as schwertmannite and jarosite) in the associated active layers and along perennial/intermittent “rusting” streams/rivers.^29,30,113^ Given the active roles of these secondary Fe(III) phases in retaining and stabilizing OM under acidic sulfate-rich conditions on our AS soil field, they likely exert a strong influence on the distribution and stability of the vast amounts of perennially frozen OM pools being remobilized from the active layers.

## Supporting Information Available

Detailed procedures for extracting and measuring hot-water-extractable OC, acid-hydrolyzable OC, and hydroxyl radicals and for collecting and processing iron XAS data; key features of the sample XAS spectra; interpretation of weight loss peaks in sample TGA curves; physiochemical properties of the samples from the study field; summary of the Fe reference compounds; data on the abundances of hydroxy radicals, pH of hot-water extracts, concentrations of two organic fractions and total OC and N in selected samples; photograph showing one sampling soil trench; calibration curve of fluorescent dihydroxyterephthalic acid (HTPA); iron first-derivative XANES and *k*^3^-weighted EXAFS spectra; plots of 1 M HCl-extractable Fe(III) versus 1 M HCl-extractable DOC and TOC/TN atomic ratios; and histograms and normal probability plots for the standardized residuals of linear regression models (PDF)
