# Supplementary material for: Storage and Distribution of Organic Carbon and Nutrients in Acidic Soils Developed on Sulfidic Sediments: The Roles of Reactive Iron and Macropores
Source: Environ Sci Technol. 2024 May 14;58(21):9200–12. doi: 10.1021/acs.est.3c11007 (PMC11137870; doi:10.1021/acs.est.3c11007)
Supplement: Supplementary file 1 — es3c11007_si_001.pdf [file es3c11007_si_001.pdf]

Supplementary information (SI) for:

Storage and distribution of organic carbon and nutrients in acidic soils developed on sulfidic sediments: the roles of reactive iron and macropores

Changxun Yu[a*], Nguyen Tan Luong[b], Mohammed E. Hefni[c], Zhaoliang Song[d], Eva Högfors-Rönnholm[e], Sten Engblom[e], Shurong Xie[f], Roman Chernikov[g], Markus Broström[h], Jean-François Boily[b], Mats E. Åström[a]

[a]*Department of Biology and Environmental Science, Linnaeus University, 39231 Kalmar, Sweden*
[b]*Department of Chemistry, Umeå University, 90187 Umeå, Sweden*
[c]*Department of Chemistry and Biomedical Sciences, Linnaeus University, 39231 Kalmar, Sweden*
[d]*Institute of Surface-Earth System Science, School of Earth System Science, Tianjin University, Tianjin, 300072, China*
[e]*Research and Development, Novia University of Applied Sciences, 65200 Vaasa, Finland*
[f]*School of Earth Sciences, East China University of Technology, Nanchang 330013, China*
[g]*Canadian Light Source, 44 Innovation Boulevard, Saskatoon, SK S7N 2V3, Canada*
[h]*Thermochemical Energy Conversion Laboratory, Department of Applied Physics and Electronics, Umeå University, 90187 Umeå, Sweden*

[*] Corresponding author

Email address: changxun.yu@lnu.se; yuchangxun2006@163.com

Number of pages (19)

Number of Figures (9)

Number of tables (5)

Text S1: Extraction and determination of hot-water-extractable and acid-hydrolyzable organic carbon

Hot-water-extractable and acid-hydrolyzable organic fractions were extracted by shaking 0.5 g pulverized subsamples (pre-dried in N_2) in 20 mL MQ water at 45 ℃ for 8 hours (in a water bath), and in 20 mL 1 M HCl at room temperature for 4 hours, respectively. To avoid the oxidation of reduced species in the reduced-zone and transition-zone samples during the hot-water extraction, these samples were mixed with deoxygenated MQ water in Falcon tubes inside a glove box. Thereafter, the tubes were sealed with multiple layers of parafilm and Tesa tapes, before being transferred out and shaken in the water bath. The supernatant solutions were obtained by centrifugation (8000 g x 10 minutes) and then filtered through 0.45 μm polyethersulfone membrane filters. The concentrations of dissolved organic carbon (DOC) in the supernatant solutions were immediately measured with the expulsion-based spectrophotometric method using LCK 380 test kits (as described by a previous study[1]). The performance and overall accuracy of this method were cross-validated against conventional DOC determination methods.[1] Since the measurement requires an optimum pH-range of 4–10, some of the MQ extractants with pH<4 (the pH of the extracts was measured using a pre-calibrated pH meter) and all of the HCl extractants were adjusted to pH of between 4.0 and 4.5 using diluted NaOH.

Text S2: Details of high-performance liquid chromatography (HPLC) analysis

In brief, a ~0.2 g pulverized sample was mixed with 4 mL 1 mM terephthalic acid (TPA) in 15 mL Falcon tubes. The tubes were immediately wrapped by two layers of Al foil and then incubated for 15 hours on an orbital shaker at 120 rpm. Thereafter, the suspensions were filtered through 0.45 μm filters. To avoid the oxidation of reduced species, the reduced-zone and transition-zone samples were mixed with deoxygenated TPA and processed (i.e., shaking and filtration) inside a glove box (O_2 < 0.5 ppm). During the incubation, the released $OH^{\bullet}$ was trapped by TPA (nonfluorescent), forming dihydroxyterephthalic acid (HTPA, fluorescent) with a conversion factor of 6.25.[2, 3] The

formed HTPA was quantified by an HPLC system (Agilent 1100, Agilent Technologies, USA), consisting of a quaternary gradient pump, a thermostated autosampler and column compartment (10 °C), a fluorescence detector (ex/em 309/412 nm), a multiwavelength detector (290 nm) and Chemstation software (Rev A 10.02 [1757]). HTPA was separated on a Develosil C_{18} column (Develosil ODS-UG-5µm, 4.6 mm × 250 mm, Nomura Chemical Co., Japan) with a C_{18} (1 mm) guard column under linear gradient elution conditions using 100 mM K_2HPO_4 2% of KCl (pH 4.37) and acetonitrile at a flow rate of 0.4 mL/min, and an injection volume of 10 µL. Peaks were identified by retention time. Quantification was based on fluorescence detection using a multilevel (0.01–0.5, n = 5) external calibration curve (Fig. S2). A background spectrum of 1 mM TPA (blank) was subtracted from the signals of the samples.

Text S3: Iron K-edge XAS data collection

The XAS data were recorded in transmission mode at room temperature on the BioXAS-Spectroscopy main beamline (07ID-2M) of the Canadian Light Source and the Balder beamline of the MAX IV laboratory. The subsamples were finely pulverized and analyzed as pellets. On the Balder beamline, the XAS data were recorded from -200 to +570 eV relative to the Fe K-edge using a fly scan mode with a 0.5 eV step and an acquisition time of 0.5 s per step. To achieve satisfactory signal-to-noise ratios, 11–38 scans were recorded for each sample. On the BioXAS beamline, the XAS data (2–7 scans per sample) were recorded at 10 eV steps in the pre-edge region (6912–7082 eV), at 0.5 eV steps in the near-edge region (7082–7152 eV), and at 0.05 $Å^{-1}$ steps over the extended X-ray absorption fine structure (EXAFS) region, in line with a Fe metal foil for internal energy calibration. No radiation-induced damage was observed during the measurements, as individual XANES scans for each sample were identical.

Text S4: Principal component analysis (PCA) in combination with target transform (TT) testing and linear combination fitting (LCF) guided by F-test

Principal component analysis (PCA) in combination with target transform (TT) testing were applied to k^3-weighted EXAFS spectra (k-range: 2-12 $Å^{-1}$) of all samples, using SIXpack software.[4] As the first step, PCA was carried out to define the number of statistically significant components in the EXAFS dataset. The empirical indicator (IND) is supposed to be minimum when the correct number of principal components is reached.[5] However, many studies have shown that this parameter may underestimate or overestimate the actual spectral components in a dataset of EXAFS spectra.[6-8] For our EXAFS dataset, the IND reached a minimum with five principal components (Table S4). In accordance, the sixth and seventh components do not oscillate like EXAFS spectra, and thus contain little real signal and mainly noise (Figure S6). In addition, the EXAFS spectra of all samples can be reconstructed satisfactorily with the first five components (data not shown). These features and results suggest that the EXAFS dataset contains five statistically significant spectral components. Using the first five components identified by PCA, TT testing was performed to identify suitable reference spectra (Table S5). The assessment was performed based on the empirical SPOIL value: 0–1.5 excellent, 1.5–3 good, 3–4.5 fair, 4.5–6 acceptable, >6 unacceptable.[9] Based on the criteria, 13 "suitable" (SPOIL value <6) reference compounds were identified and subsequently included in linear combination fitting (LCF).

To avoid overfitting with unnecessary reference spectra, LCF was carried out under the guidance of F-tests (Hamilton tests), which allowed us to determine whether adding a new reference spectrum statistically improved the fit or not as described previously.[10] In brief, Artemis software was used to determine the number of independent data points, and the "*I*" value for LCF fit with two or more reference spectra was calculated using a regularized lower incomplete beta-function calculator. The LCF analysis was started with the reference spectrum giving the lowest R-factor (($=\Sigma(\text{data} - \text{fit})^2/\Sigma(\text{data})^2$). An additional reference spectrum giving the largest reduction in the R-factor (relative to the previous fit) was included stepwise. The total weight for all included reference spectra was not forced to 1. Only the LCF fit with "*I*" value below 0.05 was included, meaning that, relative to the previous fit (n = 1, 2,…, where n is the total number of the reference spectra used in

LCF), the addition of the new spectrum (*n+1*) giving the lowest R-factor statistically improved the fit at a 95% confidence level.

Text S5: Key features of the sample XANES and EXAFS spectra

The Fe X-ray absorption near-edge structure (XANES) and EXAFS spectra displayed distinctive features within the system (Figures S3 and S4). The 1st derivative XANES spectra of the samples from the macropore interiors and the reduced zone displayed three sharp peaks at similar energies (~7121 eV, ~7124 eV, and ~7129 eV) as the two Fe(II)-rich silicate references: hornblende and biotite (Figure S3a, b), which belong to the mica and amphibole groups, respectively, and were identified by previous semi-quantitative X-ray diffraction analysis on the soils from the study area.[17] The EXAFS spectra of these samples also displayed weak but noticeable shoulders at k = ~5.1 Å^{-1}, coinciding with the positions of the sharp sub-peaks in the EXAFS spectra of these two silicate references (Figure S4a, b). For the samples from the macropore surfaces and the ditch, the peaks at ~7121 eV and k = ~5.1 Å^{-1} were very weak or non-observable (Figures S3c and S4d). These spectral features collectively indicate that the macropore interiors and the reduced zone contained consistently higher fractional amounts of Fe(II)-rich primary minerals (e.g., hornblende and/or biotite) than the macropore surfaces and ditch precipitates.

The transition-zone samples had several spectral features that did not appear in the other samples (Figure S4b). The EXAFS oscillation peaks at $k > 5$ Å^{-1} were split into multiple sub-peaks, occurring at similar positions as those of the pyrite standard (Figure S4a, b), and the 1st derivative XANES spectra, although bearing the three characteristic peaks as the biotite and hornblende references, displayed a sub-peak at ~7118 eV, matching the main peak in the spectrum of the pyrite (Figure S3a,b). These features provide strong evidence that, in addition to the Fe(II)-rich primary minerals, considerable fractions of the Fe in the transition zone occurred as pyrite.

Text S6: Interpretation of weight-loss peaks in the TGA curves of three samples from the macropore surfaces and corresponding interior counterparts, plus one sample from the reduced zone

Due to the loss of surface-absorbed or free water molecules trapped within mineral/organic aggregates, the weights of all the samples displayed an abrupt drop between 50 and 180 °C, with a large derivative weight-loss peak centering around 100 °C. Previous temperature-programmed desorption experiments also revealed a quick removal of singly coordinated OH groups at the surfaces of Fe hydroxides and schwertmannite at temperatures below 125 °C.[11, 12] Given the widespread occurrence of these Fe phases in macropores (both surfaces and interiors), these low-temperature dehydroxylation processes should have contributed to the first weight-loss peak of the six samples from macropore surfaces and interior counterparts. All the samples also displayed strong weight-loss peaks around 270–280 °C. Within this temperature interval, only very low quantities of organic carbon in these samples were volatilized and combusted to CO_2. Thus, these weight-loss peaks were not linked to OC combustion, but mainly contributed by the dehydration and dehydroxylation of structural H_2O and OH groups in secondary minerals (e.g., Al/Fe hydroxides and schwertmannite) that have been shown to occur at these temperatures.[12, 13] The latter processes are further supported by the fact that the three samples from the macropore surfaces with high amounts of Fe hydroxides and oxyhydroxysulfates had overall greater weight loss at these temperatures. In addition, the three samples from the macropore surfaces displayed one broad weight-loss peak between 600 and 700 °C. Since the removal of structural sulfate (as SO_2 (g) or SO_3 (g)) was previously found to occur at a similar temperature range (520–700 °C)[13] and these samples contained abundant 1 M HCl-extractable S (0.17-0.41%, Table S1) likely largely bound to schwertmannite, these peaks were assigned to loss of structural sulfate primarily bound to schwertmannite. The reduced-zone sample displayed three additional notable peaks, centering around 480 °C, 770 °C, and 880 °C. These temperatures match those of the weight-loss and SO_2 concentration peaks reported for pyrrhotite.[14] Given that the reduced zone contains both pyrite and

iron monosulfides (e.g., FeS), as shown in this study and previous research,[15, 16] these additional peaks most likely reflect thermal decomposition of iron-sulfide minerals.

Table S1. The pH and Eh (mV) values and concentrations (%) of 1 M HCl-extractable S, Fe(II), Fe(III), and P in all soil samples and two ditch precipitates from the acid sulfate (AS) soil field.

Site	Sampling location	Sample	pH	Eh	S	Fe(II)	Fe(III)	P	Fe XAS
A	Macropore interiors	$A_{\text{-int-1}}$			0.02	0.05	0.42	0.09	Yes
		$A_{\text{-int-2}}$			0.03	0.06	0.41	0.05	Yes
		$A_{\text{-int-3}}$			0.04	0.06	0.56	0.08	Yes
	Macropore surfaces	$A_{\text{-surf-1}}$	3.7	746	0.41	0.10	5.08	0.19	Yes
		$A_{\text{-surf-2}}$	3.7	749	0.49	0.11	5.15	0.09	Yes
		$A_{\text{-surf-3}}$	3.6	754	0.49	0.11	6.42	0.28	Yes
	Reduced zone	$A_{\text{-red-1}}$			1.20	0.94	0.00	0.07	Yes
		$A_{\text{-red-2}}$			1.33	1.04	0.00	0.07	Yes
B	Macropore interiors	$B_{\text{-int-1}}$			0.03	0.06	0.48	0.10	
		$B_{\text{-int-2}}$			0.04	0.06	0.58	0.07	
		$B_{\text{-int-3}}$			0.04	0.05	0.36	0.06	
	Macropore surfaces	$B_{\text{-surf-1}}$	3.7	747	0.49	0.12	6.70	0.26	
		$B_{\text{-surf-2}}$	3.7	745	0.37	0.08	3.82	0.05	
		$B_{\text{-surf-3}}$	3.6	751	0.28	0.10	3.39	0.10	
C	Macropore interiors	$C_{\text{-int-1}}$			0.03	0.07	0.58	0.08	
		$C_{\text{-int-2}}$			0.10	0.09	1.45	0.12	
		$C_{\text{-int-3}}$			0.03	0.09	0.29	0.05	
	Macropore surfaces	$C_{\text{-surf-1}}$	3.8	733	0.34	0.12	4.49	0.19	
		$C_{\text{-surf-2}}$	3.8	734	0.40	0.08	5.36	0.27	
		$C_{\text{-surf-3}}$	3.7	738	0.15	0.09	1.47	0.09	
D	Macropore interiors	$D_{\text{-int-1}}$			0.12	0.09	1.33	0.08	
		$D_{\text{-int-2}}$			0.11	0.09	1.42	0.08	
		$D_{\text{-int-3}}$			0.04	0.06	0.60	0.05	
	Macropore surfaces	$D_{\text{-surf-1}}$	3.7	703	0.37	0.12	3.87	0.09	
		$D_{\text{-surf-2}}$	3.6	654	0.26	0.10	2.94	0.08	
		$D_{\text{-surf-3}}$	3.6	706	0.27	0.10	2.99	0.09	
E	Macropore interiors	$E_{\text{-int-1}}$			0.07	0.07	0.64	0.04	
		$E_{\text{-int-2}}$			0.19	0.10	1.69	0.09	
		$E_{\text{-int-3}}$			0.19	0.11	1.80	0.09	
	Macropore surfaces	$E_{\text{-surf-1}}$	3.4	634	0.26	0.10	2.44	0.07	
		$E_{\text{-surf-2}}$	3.6	679	0.34	0.11	3.33	0.08	
		$E_{\text{-surf-3}}$	3.6	727	0.34	0.11	3.10	0.05	
F	Macropore interiors	$F_{\text{-int-1}}$			0.08	0.08	0.87	0.08	
		$F_{\text{-int-2}}$			0.09	0.15	0.83	0.06	
		$F_{\text{-int-3}}$			0.04	0.09	0.57	0.09	Yes
	Macropore surfaces	$F_{\text{-surf-1}}$	3.9	681	0.20	0.12	2.00	0.09	
		$F_{\text{-surf-2}}$	3.8	731	0.19	0.12	2.02	0.10	
		$F_{\text{-surf-3}}$	3.8	741	0.29	0.13	3.04	0.12	Yes
	Transition zone	$F_{\text{-trans-1}}$			0.02	0.32	0.00	0.05	Yes

G	Macropore interiors	$G_{\text{-int-1}}$			0.16	0.11	1.66	0.13	
		$G_{\text{-int-2}}$			0.07	0.09	0.77	0.10	
		$G_{\text{-int-3}}$			0.06	0.09	0.64	0.07	
	Macropore surfaces	$G_{\text{-surf-1}}$	4.0	701	0.23	0.12	2.60	0.17	
		$G_{\text{-surf-2}}$	3.9	711	0.47	0.14	4.79	0.16	
		$G_{\text{-surf-3}}$	3.8	721	0.28	0.13	3.12	0.13	
H	Macropore interiors	$H_{\text{-int-1}}$			0.03	0.09	0.51	0.07	
		$H_{\text{-int-2}}$			0.11	0.10	1.45	0.08	
		$H_{\text{-int-3}}$			0.09	0.09	0.94	0.08	
	Macropore surfaces	$H_{\text{-surf-1}}$	4.8	701	0.20	0.11	2.06	0.09	
		$H_{\text{-surf-2}}$	3.9	731	0.34	0.13	3.42	0.07	
		$H_{\text{-surf-3}}$	4.0	731	0.29	0.12	3.06	0.08	
I	Macropore interiors	$I_{\text{-int-1}}$			0.19	0.13	2.14	0.11	Yes
		$I_{\text{-int-2}}$			0.08	0.09	0.93	0.07	
		$I_{\text{-int-3}}$			0.29	0.14	3.34	0.13	Yes
	Macropore surfaces	$I_{\text{-surf-1}}$	3.8	741	0.26	0.12	2.53	0.09	Yes
		$I_{\text{-surf-2}}$	3.8	741	0.24	0.12	2.58	0.10	
		$I_{\text{-surf-3}}$	3.7	711	0.42	0.13	4.26	0.11	Yes
J	Macropore interiors	$J_{\text{-int-1}}$			0.05	0.09	0.73	0.07	Yes
		$J_{\text{-int-2}}$			0.19	0.12	2.29	0.11	
		$J_{\text{-int-3}}$			0.16	0.11	1.64	0.08	
	Macropore surfaces	$J_{\text{-surf-1}}$	3.9	586	0.37	0.14	4.03	0.09	Yes
		$J_{\text{-surf-2}}$	3.8	721	0.30	0.13	3.38	0.12	
		$J_{\text{-surf-3}}$	3.7	691	0.29	0.12	3.07	0.10	
	Transition zone	$J_{\text{-trans-1}}$			0.01	0.31	0.00	0.06	Yes
K	Macropore interiors	$K_{\text{-int-1}}$			0.04	0.08	0.57	0.06	
		$K_{\text{-int-2}}$			0.13	0.10	1.36	0.08	
		$K_{\text{-int-3}}$			0.11	0.11	1.22	0.09	
	Macropore surfaces	$K_{\text{-surf-1}}$	4.3	711	0.13	0.11	1.53	0.08	
		$K_{\text{-surf-2}}$	4.2	711	0.25	0.13	2.69	0.10	
		$K_{\text{-surf-3}}$	4.0	721	0.25	0.13	2.72	0.12	
Ditch		$\text{Ditch}_{\text{-precip-1}}$			0.66	0.10	7.48	0.17	Yes
		$\text{Ditch}_{\text{-precip-2}}$			0.43	0.06	4.85	0.06	Yes

Note: The subscripts "surf" and "int" refer to macropore surface and interior, respectively, while "red", "trans", and "precip" refer to reduced zone, transition zone, and ditch precipitate, respectively. The subscript numbers mark the three sub-zones (Figure S1) at each sampling trench.

Table S2. Summary of the Fe reference compounds included in the study.

Reference	Group	Origin	Beamline facility	Data sources
Chlorite	Phyllosilicate	Natural[a]	I811_MAX-lab	Yu et al. (2020)[17]
Biotite	Phyllosilicate	Natural[a]	I811_MAX-lab	Yu et al. (2020)[17]
Hornblende	Inosilicate	Natural[a]	I811_MAX-lab	Yu et al. (2020)[17]
Illite	Phyllosilicate	Natural[a]	I811_MAX-lab	Yu et al. (2020)[17]
Muscovite	Phyllosilicate	Natural[a]	I811_MAX-lab	Yu et al. (2020)[17]
Schwertmannite	Ferric oxyhydrosulfate	Synthetic	Balder_MAX-IV	Shahabi-Ghahfarokhi et al. (2022)[18]
Jarosite	Ferric oxyhydrosulfate	Synthetic	Balder_MAX-IV	Shahabi-Ghahfarokhi et al. (2022)[18]
2-line ferrihydrite	Ferric hydroxide	Synthetic	Balder_MAX-IV	Shahabi-Ghahfarokhi et al. (2022)[18]
Goethite	Ferric hydroxide	Synthetic	Balder_MAX-IV	Shahabi-Ghahfarokhi et al. (2022)[18]
Lepidocrocite	Ferric hydroxide	Synthetic	Balder_MAX-IV	Shahabi-Ghahfarokhi et al. (2022)[18]
Hematite	Ferric oxide	Natural[b]	I811_MAX-lab	Yu et al. (2020)[17]
Magnetite	Mixed-valence Fe oxide	Synthetic	Balder_MAX-IV	Shahabi-Ghahfarokhi et al. (2022)[18]
Fe(III)-sorbed	Organically complexed Fe(III)	Synthetic	I811_MAX-lab	Yu et al. (2015)[19]
Fe(II)-sorbed	Organically complexed Fe(II)	Synthetic	I811_MAX-lab	Yu et al. (2015)[19]
Aqueous Fe(II)	Outer-sphere Fe(II) complexes	Synthetic	I811_MAX-lab	Yu et al. (2015)[19]
Siderite	Ferrous carbonate	Synthetic	17C_National Synchrotron	Burton et al. (2012)[20]
FeS	Iron sulfide	Synthetic	Balder_MAX-IV	Shahabi-Ghahfarokhi et al. (2022)[18]
Pyrite	Iron sulfide	Synthetic	I811_MAX-lab	Yu et al. (2015)[19]

[a] Isolated from granitoidic fracture networks in Laxemar, Southern Sweden.
[b] Hematite ore provided by the Department of Earth Sciences, University of Gothenburg (Origin: Marble Bar, Western Australia).

Table S3. Abundances of hydroxy radicals, pH of hot-water extracts, concentrations of hot-water- and 1 M HCl-extractable DOC, contents of total organic carbon (TOC) and total nitrogen (TN), and atomic TOC/TN ratios for selected soil samples from the AS soil field.

	Sample	Hydroxy radicals (nmol/kg)	pH of hot water extracts	Hot-water-extractable DOC (%)	1 M HCl-extractable DOC (%)	TOC (%)	Percentage of hot-water-extractable DOC (%)	Percentage of 1 M HCl-extractable DOC (%)	TN(%)	Atomic TOC/TN ratios
Macropore interiors	A_{-int-1}	564±14	4.63	0.08	0.64	1.5	5.3	43	0.3	5.8
	A_{-int-2}	637±10	4.42	0.06	0.57	1.6	3.8	36	0.3	6.2
	A_{-int-3}	627±14	4.59	0.07	0.41	1.6	4.4	26		
	B_{-int-1}	703±10	4.84	0.06	0.61	1.5	4.0	41		
	B_{-int-2}	640±65	4.76	0.07	0.58	1.5	4.7	39	0.3	5.8
	B_{-int-3}	670±31	4.86	0.06	0.60	1.5	4.0	40		
	C_{-int-1}	725±5	4.83	0.14	0.50	1.6	8.8	31	0.3	6.2
	F_{-int-1}	665±39	4.53	0.17	0.64	1.2	14	53	0.3	4.7
	F_{-int-2}	598±25	6.12	0.15	0.83	1.3	12	64	0.3	5.1
	F_{-int-3}	707±65	4.86	0.06	0.70	1.3	4.6	54		
	G_{-int-1}	710±49	4.86	0.05	0.62	1.4	3.6	44		
	G_{-int-2}	664±23	4.63	0.05	0.58	1.3	3.8	45	0.3	5.1
	I_{-int-1}	671±30	4.62	0.20	0.71	1.4	14	51		
	I_{-int-2}	718±50	4.53	0.04	0.68	1.4	2.9	49		
	I_{-int-3}	616±19	4.54	0.05	0.68	1.4	3.6	49		
	J_{-int-1}	698±9	4.75	0.04	0.55	1.1	3.6	50	0.3	4.3
	K_{-int-3}	710±13	4.68	0.12	0.86	1.3	9.2	66		
Macropore surfaces	$A_{-surf-1}$	557±13	4.04	0.05	1.14	1.2	4.2	95	0.3	4.7
	$A_{-surf-2}$	546±0	3.85	0.03	0.79	0.9	3.3	88	0.3	3.5
	$A_{-surf-3}$	586±26	4.00	0.04	0.80	1.1	3.6	73		
	$B_{-surf-1}$	608±20	4.15	0.03	0.80	0.9	3.3	89		
	$B_{-surf-2}$	593±13	4.11	0.04	0.85	1.1	3.6	77	0.3	4.3
	$B_{-surf-3}$	600±14	4.31	0.04	0.78	1.1	3.6	71		
	$C_{-surf-1}$	610±20	4.36	0.03	0.67	1.5	2.0	45	0.3	5.8
	$A_{-surf-1}$	654±7	4.59	0.18	0.62	1.1	16	56	0.3	4.3
	$F_{-surf-2}$	617±3	5.66	0.09	0.79	1.1	8.2	72	0.3	4.3

	F-surf-3	621±2	4.25	0.03	0.54	1.1	2.7	49		
	G-surf-1	613±20	4.38	0.04	0.88	1.1	3.6	80		
	G-surf-2	594±14	4.24	0.03	0.72	1.1	2.7	65	0.3	4.3
	I-surf-1	619±1	4.37	0.04	0.75	1.3	3.1	58		
	I-surf-2	611±1	4.43	0.05	0.65	1.3	3.8	50		
	I-surf-3	549±11	4.27	0.04	0.71	1.1	3.6	65		
	J-surf-1	626±2	4.47	0.04	0.70	1.1	3.6	64	0.3	4.3
	K-surf-3	634±1	4.58	0.06	0.84	1.0	6.0	84		
Transition zone	F-trans-1	738±38	6.18	0.08	0.62	1.3	6.2	48	0.4	3.8
	J-trans-1	672±13	6.05	0.10	0.57	1.6	6.3	36	0.4	4.7
Reduced zone	A-red-1	643±94	6.54	0.17	0.58	1.6	11	36	0.4	4.7
	A-red-2	675±9	6.71	0.13	0.72	1.4	9.3	51	0.3	5.4

Table S4. PCA output parameters for Fe EXAFS spectra.

	Eigen values	Cum Var	IND
**C1**	**108.3**	**50.3**	**0.02256**
**C2**	**29.2**	**67.2**	**0.01468**
**C3**	**11.1**	**72.6**	**0.01417**
**C4**	**9.1**	**77.1**	**0.01387**
**C5**	**7.7**	**80.9**	**0.01370**
C6	6.0	83.8	0.01405
C7	5.8	86.7	0.01395

Table S5. Results of target transformation testing on the reference spectra using the first five principal components extracted from the EXAFS spectra.

Reference spectra	SPOIL	R-factor[a]	Suitability[b]
Biotite	1.53	0.0662	Good
Jarosite	1.66	0.0514	Good
Hornblende	1.74	0.0632	Good
2-line ferrihydrite	2.06	0.0360	Good
Pyrite	2.70	0.2159	Good
Chlorite	2.80	0.1345	Good
Schwertmannite	4.04	0.0927	Fair
Aqueous Fe(II)	4.39	0.2041	Fair
Illite	4.59	0.0498	Acceptable
Goethite	4.67	0.2291	Acceptable
Fe(III)-sorbed humic	5.31	0.0951	Acceptable
Fe(II)-sorbed humic	5.99	0.2241	Acceptable
FeS	5.86	0.4351	Acceptable
Hematite	6.08	0.3444	Unacceptable
Muscovite	7.10	0.1674	Unacceptable
Magnetite	8.13	0.5827	Unacceptable
Lepidocrocite	8.46	0.3072	Unacceptable
Siderite	12.3	0.6502	Unacceptable

[a]R-factor=$\sum((\text{data} - \text{fit})^2/\sum data^2)$, [b]Suitability was classified based on the SPOIL values by Malinowski 1978: 0–1.5 excellent, 1.5–3 good, 3–4.5 fair, 4.5–6 acceptable, >6 unacceptable.

Figure S1. A photograph showing the different soil zones in an excavated trench at one site in the AS soil field. After being excavated, the trench was immediately sub-divided into three sub-zones for sampling the precipitates on the surfaces of macropores and clayish materials in the macropore interiors. The macropores in the acidic zone mainly consist of interconnected cracks, fissures, and tubular pores are covered by massive brownish-to-yellowish precipitates, while the macropore interiors refer to the clayish materials underneath these precipitates. Although the structure and physical properties of the macropores might have been partly disturbed/altered during the

excavation, the mineral precipitates on the surfaces of the macropores and their underlying clayish materials should remain intact.

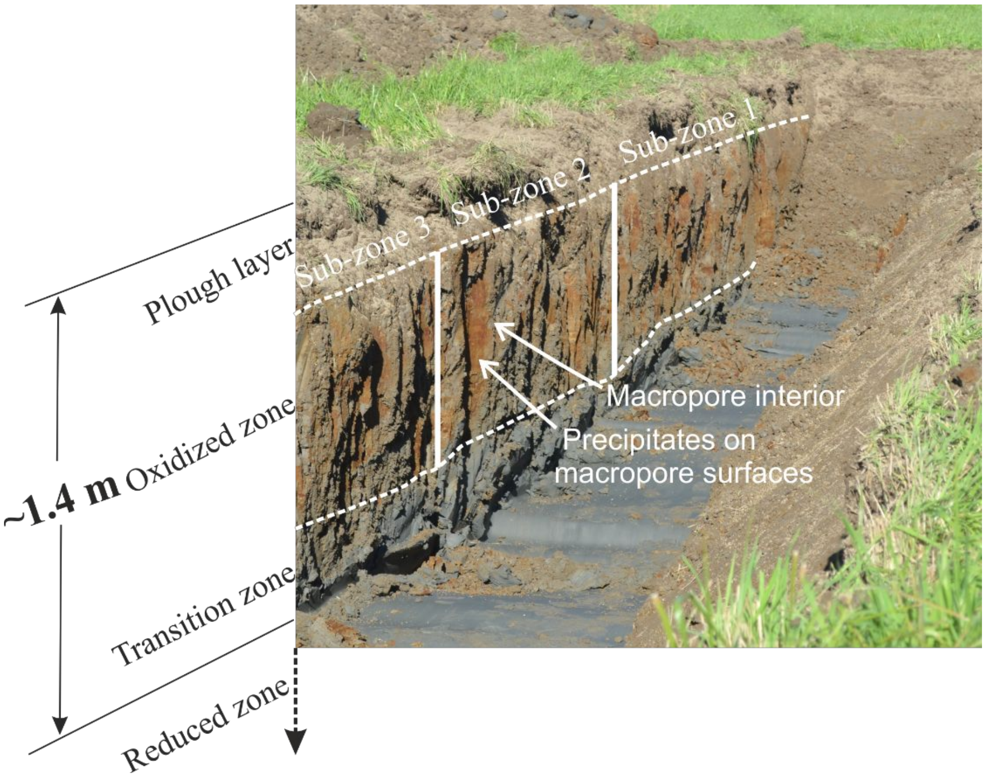

Figure S2. Calibration curve of fluorescent dihydroxyterephthalic acid (HTPA). The grey lines represent 95% confidence intervals for the best linear fit.

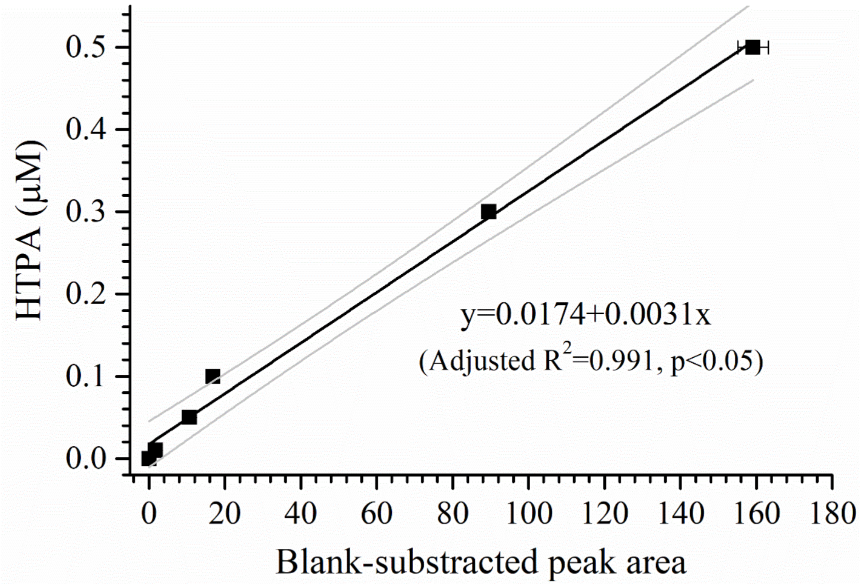

Figure S3. Iron K-edge first-derivative XANES spectra for selected reference materials (a), and soil samples from the macropore surfaces and corresponding interior counterparts at seven sites (b, c). The spectra of the samples from the transition and reduced zones, as well as those of the ditch precipitates, were also plotted for comparison.

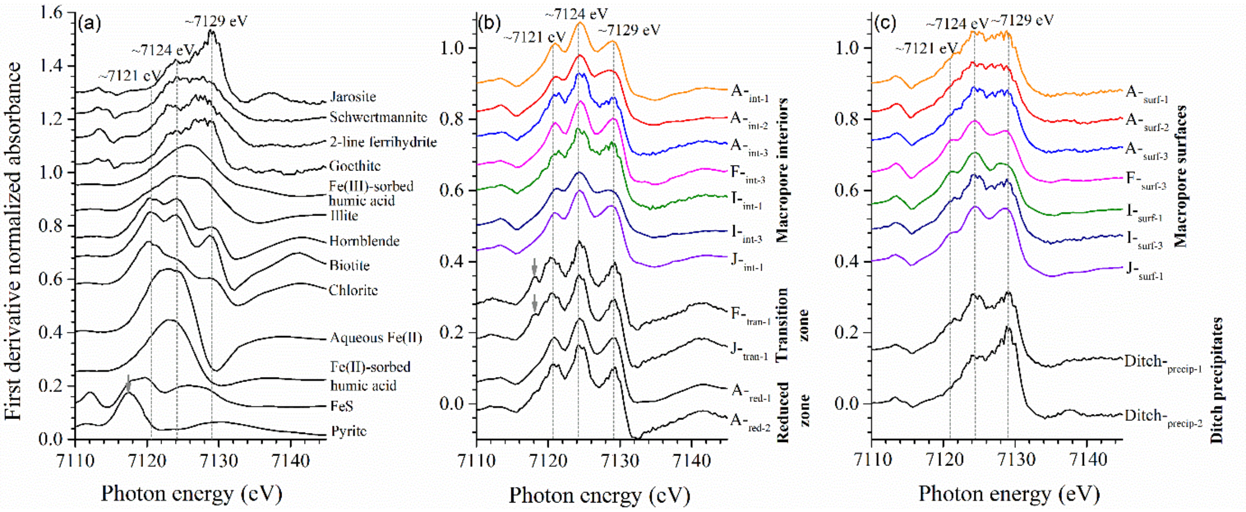

Figure S4. Iron K-edge EXAFS spectra of the references included in the LCF, compared to those of soil samples from macropore surfaces and corresponding interior counterparts at seven sites. Also shown are spectra of the samples from the transition zone, reduced zone, and ditch.

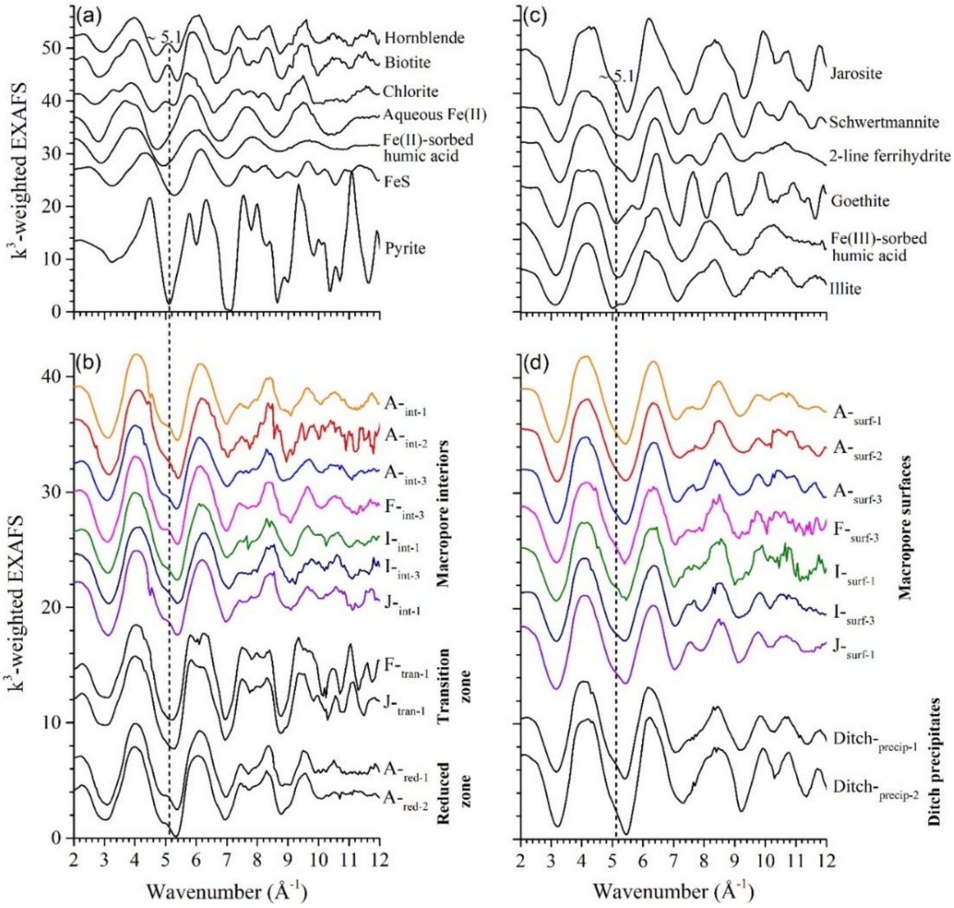

Figure S5. Correlations between the concentrations of 1 M HCl-extractable Fe(III) and DOC (a) and between the concentrations of 1 M HCl-extractable Fe(III) and atomic ratios of TOC/TN (b) in selected soil samples from the AS soil field.

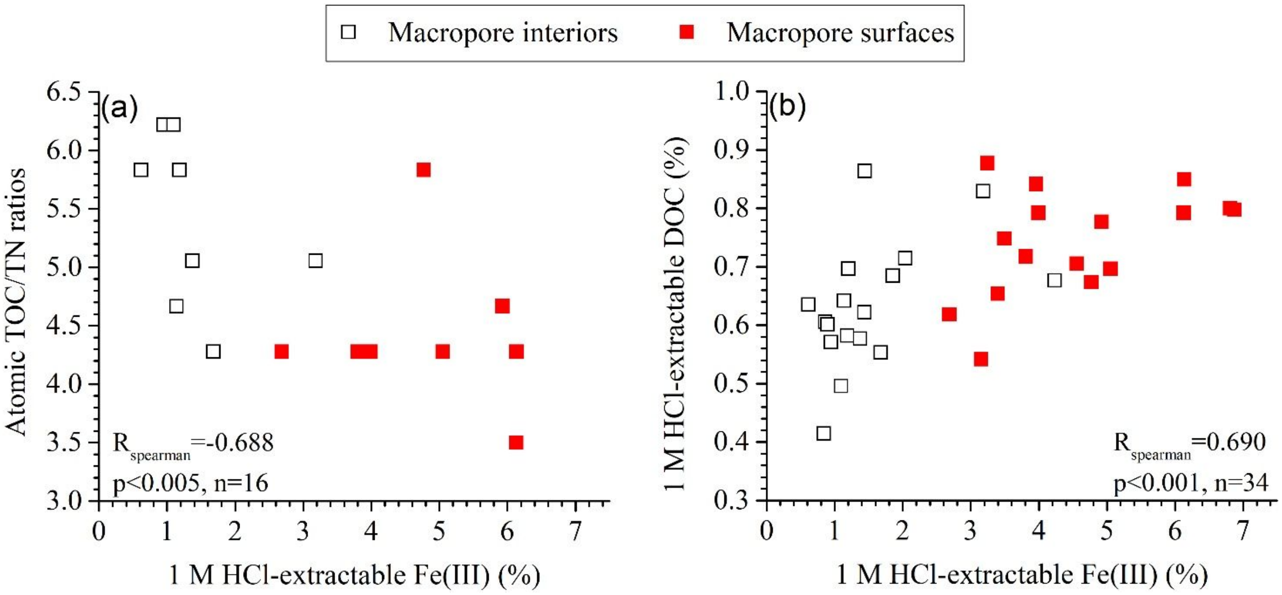

Figure S6. The first seven principal components, as determined by PCA applied to the EXAFS spectra of selected samples from the acid sulfate soil farmland. For clarity, the amplitudes of components PC2–PC7 were multiplied by the factors next to each line in the figure.

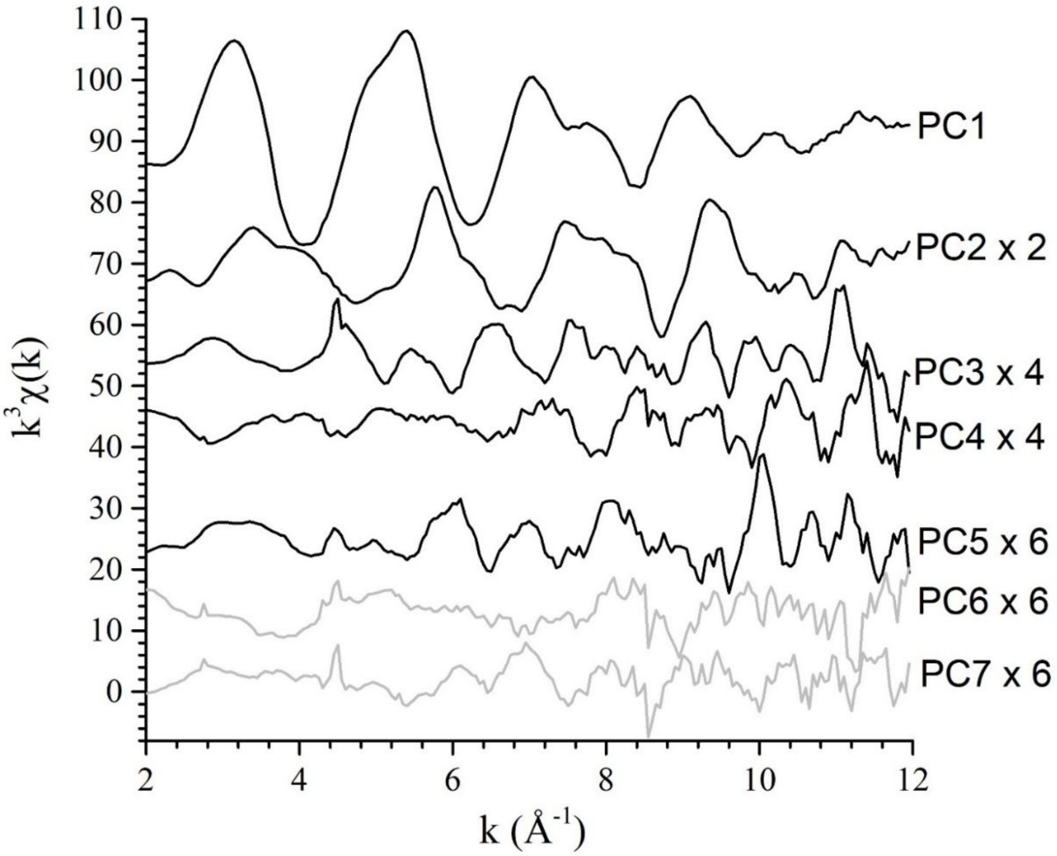

Figure S7. Histogram and normal probability plot for the standardized residuals of the linear regression model in Figure 1a.

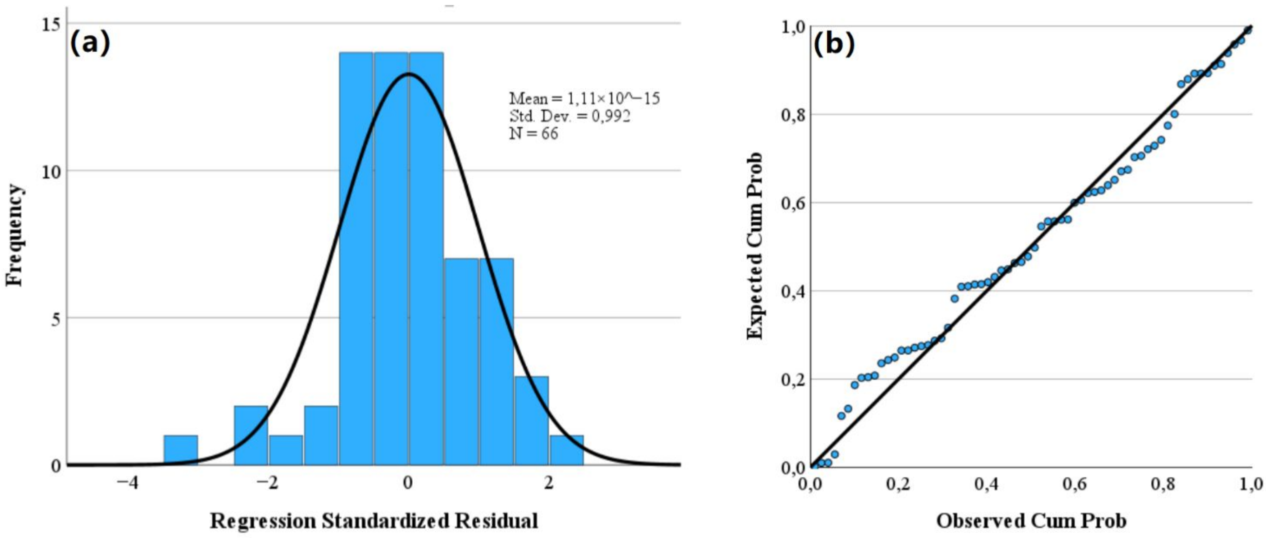

Figure S8. Histogram and normal probability plot for the standardized residuals of the linear regression fit to the concentrations of 1 M HCl -extractable P and Fe(III) on the macropore surfaces (Figure 1b).

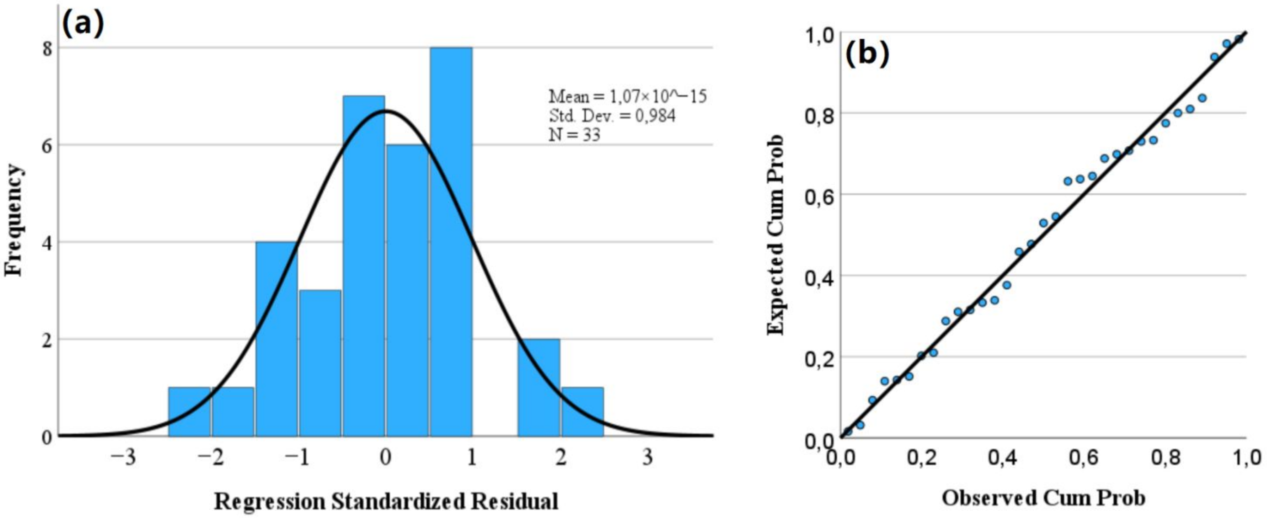

Figure S9. Histogram and normal probability plot for the standardized residuals of the linear regression fit to the concentrations of 1 M HCl -extractable P and Fe(III) in the macropore interiors (Figure 1b).

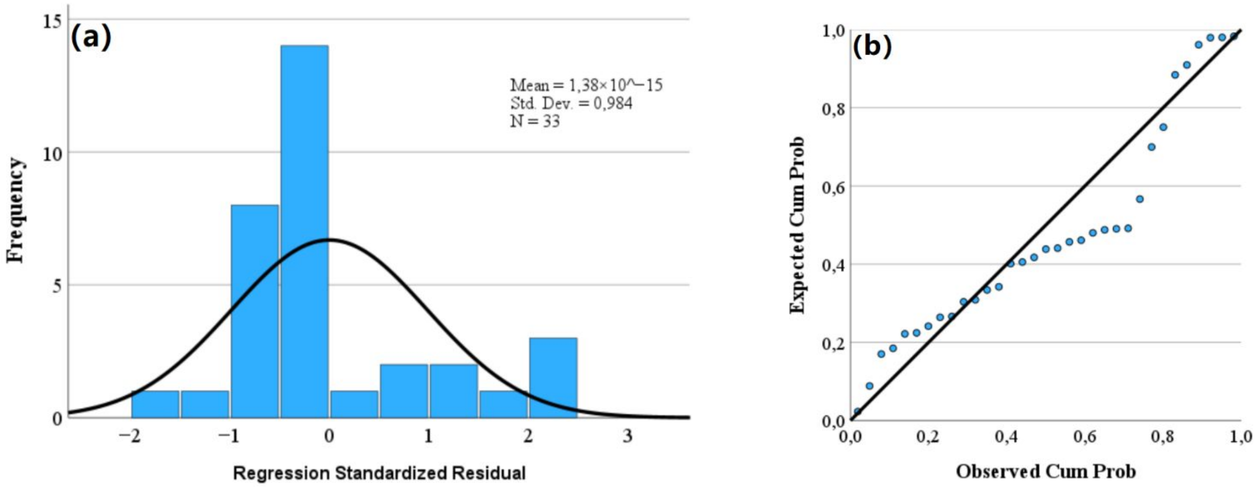

References

1. Avagyan, A.; Runkle, B. R.; Kutzbach, L., Application of high-resolution spectral absorbance measurements to determine dissolved organic carbon concentration in remote areas. *J. Hydrol.* **2014,** *517*, 435-446.

2. Li, L.; Tomonori, A.; Yukiko, N.; Risa, K.; Noriko, U.; Kazuhiro, I.; Tadahiko, M.; Masataka, M.; Naoki, M., An HPLC assay of hydroxyl radicals by the hydroxylation reaction of terephthalic acid. *Biomed. Chromatogr.* **2004,** *18*, (7), 470-4.

3. Georgiou, C. D.; Sun, H. J.; McKay, C. P.; Grintzalis, K.; Papapostolou, I.; Zisimopoulos, D.; Panagiotidis, K.; Zhang, G.; Koutsopoulou, E.; Christidis, G. E.; Margiolaki, I., Evidence for photochemical production of reactive oxygen species in desert soils. *Nat. Commun.* **2015,** *6*, 7100.

4. Webb, S., SIXpack: a graphical user interface for XAS analysis using IFEFFIT. *Physica. scripta.* **2005,** *2005*, (T115), 1011.

5. Malinowski, E. R., Determination of the number of factors and the experimental error in a data matrix. *Anal. Chem.* **1977,** *49*, (4), 612-617.

6. Kirpichtchikova, T. A.; Manceau, A.; Spadini, L.; Panfili, F.; Marcus, M. A.; Jacquet, T., Speciation and solubility of heavy metals in contaminated soil using X-ray microfluorescence, EXAFS spectroscopy, chemical extraction, and thermodynamic modeling. *Geochim. Cosmochim. Acta.* **2006,** *70*, (9), 2163-2190.

7. Sarret, G.; Balesdent, J.; Bouziri, L.; Garnier, J.-M.; Marcus, M. A.; Geoffroy, N.; Panfili, F.; Manceau, A., Zn speciation in the organic horizon of a contaminated soil by micro-X-ray fluorescence, micro-and powder-EXAFS spectroscopy, and isotopic dilution. *Environ. Sci. Technol.* **2004,** *38*, (10), 2792-2801.

8. Panfili, F.; Manceau, A.; Sarret, G.; Spadini, L.; Kirpichtchikova, T.; Bert, V.; Laboudigue, A.; Marcus, M. A.; Ahamdach, N.; Libert, M.-F., The effect of phytostabilization on Zn speciation in a dredged contaminated sediment using scanning electron microscopy, X-ray fluorescence, EXAFS spectroscopy, and principal components analysis. *Geochim. Cosmochim. Acta.* **2005,** *69*, (9), 2265-2284.

9. Malinowski, E. R., Theory of error for target factor analysis with applications to mass spectrometry and nuclear magnetic resonance spectrometry. *Anal. Chim. Acta.* **1978,** *103*, (4), 339-354.

10. Siebecker, M. G.; Chaney, R. L.; Sparks, D. L., Nickel speciation in several serpentine (ultramafic) topsoils via bulk synchrotron-based techniques. *Geoderma.* **2017,** *298*, 35-45.

11. Boily, J.-F.; Gassman, P. L.; Peretyazhko, T.; Szanyi, J.; Zachara, J. M., FTIR spectral components of schwertmannite. *Environ. Sci. Technol.* **2010,** *44*, (4), 1185-1190.

12. Song, X.; Boily, J. F., Surface and Bulk Thermal Dehydroxylation of FeOOH Polymorphs. *J. Phys. Chem. A.* **2016,** *120*, (31), 6249-57.

13. Lázaro, C.; Antelo, J.; Carabante, I.; Otero-Fariña, A.; Verdes, P. V.; Dacunha-Marinho, B.; Fiol, S., Thermal Transformation of Natural Schwertmannite in the Presence of Chromium. *Minerals.* **2022,** *12*, (6): 726.

14. Yu, D.; Liu, F.; Zhang, J.; Barati, M., Thermal Upgrading of Nickeliferous Pyrrhotite Tailings for the Recovery of Nickel in the Form of Ferronickel Alloy. *Metall. Mater. Trans. B.* **2019,** *50*, (5), 2186-2196.

15. Boman, A.; Fröjdö, S.; Backlund, K.; Åström, M. E., Impact of isostatic land uplift and artificial drainage on oxidation of brackish-water sediments rich in metastable iron sulfide. *Geochim. Cosmochim. Acta.* **2010,** *74*, (4), 1268-1281.

16. Nordmyr, L.; Boman, A.; Åström, M.; Österholm, P., Estimation of leakage of chemical elements from boreal acid sulphate soils. *Boreal. Environ. Res.* **2006,** *11*, 261.

17. Yu, C.; Drake, H.; Dideriksen, K.; Tillberg, M.; Song, Z.; Morup, S.; Astrom, M. E., A Combined X-ray Absorption and Mossbauer Spectroscopy Study on Fe Valence and Secondary Mineralogy

in Granitoid Fracture Networks: Implications for Geological Disposal of Spent Nuclear Fuels. *Environ. Sci. Technol.* **2020,** *54*, (5), 2832-2843.

18. Shahabi-Ghahfarokhi, S.; Åström, M.; Yu, C.; Lindquisit, T.; Djerf, H.; Kalbitz, K.; Ketzer, M., Extensive dispersion of metals from hemiboreal acid sulfate soil into adjacent drain and wetland. *Appl. Geochem.* **2022,** *136*.

19. Yu, C. X.; Virtasalo, J. J.; Karlsson, T.; Peltola, P.; Österholm, P.; Burton, E. D.; Arppe, L.; Hogmalm, J. K.; Ojala, A. E. K.; Åström, M. E., Iron behavior in a northern estuary: Large pools of non-sulfidized Fe(II) associated with organic matter. *Chem. Geol.* **2015,** *413*, 73-85.

20. Burton, E. D.; Johnston, S. G., Impact of silica on the reductive transformation of schwertmannite and the mobilization of arsenic. *Geochim. Cosmochim. Acta.* **2012,** *96*, 134-153.
